# Spatial and seasonal variation in macrozoobenthic density, biomass and community composition in a major tropical intertidal area, the Bijagós Archipelago, West-Africa

**DOI:** 10.1371/journal.pone.0277861

**Published:** 2022-11-28

**Authors:** Ana Pinto Coelho, Mohamed Henriques, Afonso Duarte Rocha, João Paulino, Loran Kleine Schaars, Catarina Ramos, Aissa Regalla de Barros, Teresa Catry, José Pedro Granadeiro, Theunis Piersma, José Augusto Alves

**Affiliations:** 1 Centre for Environmental and Marine Studies (CESAM), Department of Biology, University of Aveiro, Aveiro, Portugal; 2 Centre for Environmental and Marine Studies (CESAM), Department of Animal Biology, Faculdade de Ciências da Universidade de Lisboa, Lisbon, Portugal; 3 Conservation Ecology Group, Groningen Institute for Evolutionary Life Sciences, University of Groningen, Groningen, The Netherlands; 4 Conservation Biology Research Group, Department of Anatomy, Cell Biology and Zoology, University of Extremadura, Badajoz, Spain; 5 Department of Coastal Systems, NIOZ Royal Netherlands Institute for Sea Research, Texel, The Netherlands; 6 Instituto da Biodiversidade e das Áreas Protegidas Dr. Alfredo Simão da Silva (IBAP), Bissau, Guiné-Bissau; 7 South Iceland Research Centre, University of Iceland, Laugarvatn, Iceland; KAUST University, SAUDI ARABIA

## Abstract

The coastal intertidal ecosystem of the Bijagós Archipelago, Guinea-Bissau, one of the largest and most important in West Africa, sustains a considerable proportion of the migratory shorebird populations of the East Atlantic Flyway and operates as a nursery area for benthic fish in the region. The macrozoobenthos in these mudflats constitute the main food source for both groups so that spatial and temporal variation in their abundance and community composition is likely to influence the abundance and distribution of fish and birds. In this study we described the spatial and temporal dynamics in the density, biomass, and community composition of macrozoobenthos across six intertidal flats in three islands of the Bijagós Archipelago. Overall, the Bijagós Archipelago was characterised by a highly species-rich macrozoobenthic community, with ca. 88 taxa identified across all sites, reaching a mean density of 1871 ± 58.3 ind.m^-2^ (mean ± SE) and mean biomass of 5.65 ± 0.41 g of AFDM.m^-2^ (ash-free dry mass per m^2^), values much lower than what was described for nearby intertidal areas, namely the Band d’Arguin, Mauritania. Density and biomass of major macrozoobenthos classes (Bivalvia, Polychaeta, Malacostraca and Gastropoda) differed across sites and months, displaying an overall increase in density towards the final months of the dry season (March and April). Similarly, community composition also differed significantly between sites and throughout the season. The site with most distinct community composition (Adonga) supported low diversity and high abundance of a few bivalve species, whilst other two sites that hosted the most diverse communities, were also the most similar between them (Anrumai and Abu). These spatial and temporal patterns constitute an important baseline to improve knowledge of this intertidal ecosystem and will contribute towards a better understanding of the spatial and temporal distribution patterns of their consumers.

## 1. Introduction

Intertidal systems tend to be highly productive and provide numerous ecosystem services, from nutrient recycling to shoreline stabilisation and food production [1, 2]. These ecosystems are among the most widespread coastal habitats globally [3], playing an important role in the support, protection and resilience of coastal areas and their inhabitants [4–6]. Intertidal flats are threatened by global change, with sea level rise, global warming and other climatic, chemical and physical changes among the major threats, aggravated by direct anthropogenic action, like coastal development [7, 8]. These have an impact over the species assemblages in intertidal ecosystems, affecting their distribution, food and habitat availability, community composition and ultimately the entire ecosystem functioning [7, 9, 10]. It is therefore urgent to describe the current state of these systems across biological organization levels, in order to better understand future changes, particularly in understudied areas of the globe.

Intertidal flats host macrozoobenthos communities, a diverse group of species adapted to the variable environmental conditions associated with tidal regimes [11]. These communities play a fundamental role on intertidal ecosystem functioning and structure being involved in trophic and non-trophic interactions and energy transfer (e.g. [12–16], with a particularly relevant role as food source for other species in the intertidal system, such as fish and shorebirds [17–19]. The distribution and abundance of macrozoobenthos is linked to environmental conditions, such as temperature, hydrodynamic regime, sediment type and composition, primary food sources and nutrients [20–23], and to ecological characteristics, such as the presence of ecological engineers or predators [24–27]. For example, the presence of the West African fiddler crab (*Afruca tangeri*), an ecosystem engineer in intertidal sediments, has been shown to strongly limit the abundance and diversity of macrozoobenthos communities in the Bijagós Archipelago, Guinea-Bissau [28]. Similarly, exclusion experiments in a tropical intertidal flat have described the cascading effects that the removal of a predator species can have on different levels of the ecosystem, from changes in the abundance of and growth rates of prey species eventually leading to population declines, to altering the chemical processes in the mudflats [14]. The distribution and community composition of macrozoobenthos can in turn influence the distribution of its predators [28–31], which in turn can have interacting effects on the rest of the food chain [14, 32].

Compared with temperate systems, tropical intertidal flats are often characterised by a relatively higher species richness but lower density and biomass of macrozoobenthos [11, 20, 33]. Community composition in tropical regions varies greatly between sites, being highly influenced by the action of ecosystem engineers and generally having only a few abundant species, with the remaining occurring at low densities [20, 24]. Even though temperature fluctuation tends to be lower than in temperate sites, heavy rainfall during the wet season may produce substantial changes in environmental parameters, leading to marked seasonal variation in macroinvertebrate distribution and abundance [34]. However, there is currently limited information for many tropical intertidal ecosystems, particularly in West African areas, and establishing baseline knowledge on macrozoobenthos communities is important to improve our global understanding of intertidal ecosystems.

The Bijagós Archipelago, located off the West African coast, is an inverse deltaic estuary system (i.e. an estuary where salinity increases upstream), with more than 80 islands, and is surrounded by vast intertidal flats that are mostly fringed by dense mangrove forests [35, 36]. The Archipelago is one of the most important non-breeding areas for migratory shorebirds in the East Atlantic Flyway, being used by up to 10% of all the shorebirds migrating along this flyway [37–39]. It also provides refuge for important fish populations, being considered one of the major nursing areas in West Africa, and home to several species of international conservation concern [36, 40–42]. Both shorebirds and benthic fish rely on the productivity of the intertidal flats, feeding on its macrozoobenthic communities to sustain their populations. Despite the national and international recognition of the archipelago, and even though recent studies have been shedding light on the structure and functioning of its intertidal ecosystem (e.g. [28, 32, 43–45]), the spatio-temporal patterns and composition of the macrozoobenthic community remain poorly understood, with significant knowledge gaps on many groups of species and on the spatial and temporal representativity of existing studies [45, 46].

Studies on the macrozoobenthos of the Bijagós Archipelago are scarce, but recent papers reported overall low densities and biomass, with considerable variation in species composition between sites [46] and habitat partitions [28]. Nevertheless, these studies included a limited number of sites (three and one, respectively), hampering wider conclusions to the whole Bijagós Archipelago. Furthermore, little is currently known regarding how these macrozoobenthic communities vary within and between seasons, particularly during periods of increased predation pressure caused by migratory shorebirds. These shorebirds congregate in the Bijagós Archipelago from October to April [17, 37, 47], and were shown to have the potential to deplete their food stocks during their temporary stay at another West-African intertidal area, the Banc d’Arguin, in Mauritania [29]. In this study we aim to describe the overall spatial and temporal variation in the density, biomass, and community composition of macrozoobenthos in the intertidal flats of the Bijagós Archipelago, from the end of the wet season to the end of the dry season (October to April). Specifically, we investigate if the density and biomass of major macrozoobenthic classes, and the overall community composition are different between sites across the months. Furthermore, we describe which species are driving the differences observed between sites and periods (by aggregating months in three climatic and ecologically distinct periods, see methods), and also assess the differences in community diversity between sites within each period. In addition, we contribute with a set of undocumented length-biomass regressions for important macrozoobenthic taxa in the study area.

## 2. Methods

### 2.1. Study area

The Bijagós Archipelago is a group of 88 islands and islets off the coast of Guinea-Bissau, in West Africa, encompassing ca. 450 km^2^ of intertidal flats [48, 49]. Its topographical characteristics promotes large tidal amplitudes reaching up to 4.5 m in spring tides [50]. The climate is tropical and bi-seasonal, with a wet season from May to October, and a dry season from November to April. The islands are mostly surrounded by dense mangrove forests (covering ca. 699 km^2^) [36, 51] and the intertidal flats are mainly composed by bare sediments, with some areas covered by large macroalgal mats (ca. 12% of the intertidal flats) and few rocky reefs (4% of the intertidal flats) [35, 36, 49, 52]. The vast adjacent mangrove forests, the continental runoff during the rainy season, and the upwelling from the northern coast [36], contribute to biological productivity of this system in ways that remain to be discovered (but see [44, 45]).

### 2.2. Macrozoobenthos sampling

**Fig 1:**
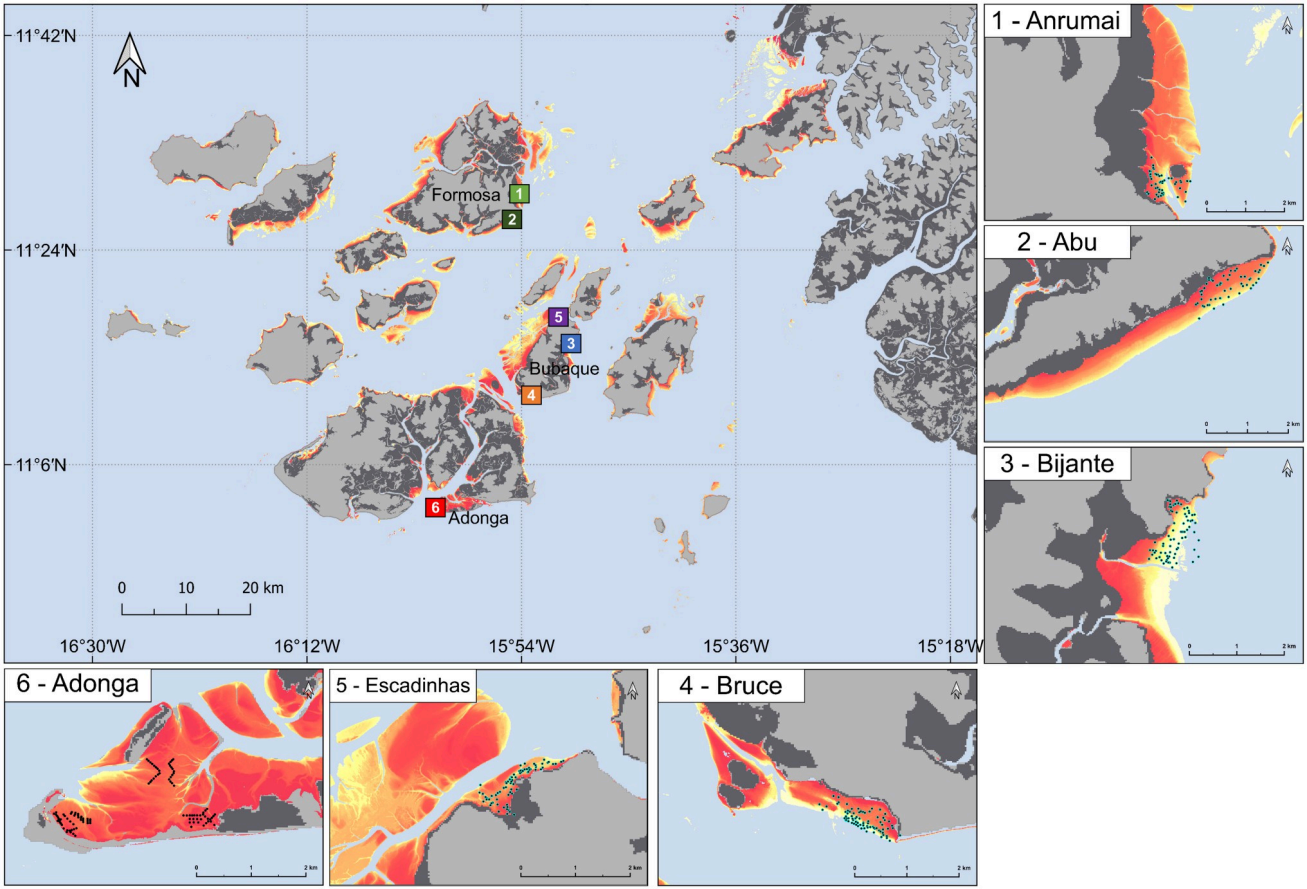
Study area, with the location of all the sampled sites (coloured squares with white numbers representing each of the sites throughout the paper). Black circles in each intertidal site (insets) represent the locations where sediment cores were taken. Note the differences in size between each intertidal site when considering the extent and distribution of sampling. Grey areas represent land, dark grey areas represent mangroves, and digital elevation model is represented for the intertidal area (ranging from yellow–orange–red showing lower, mid, and higher elevation areas).

Sampling was carried out in six sites across three islands (Fig 1): the island of Formosa, in a Marine Community Protected Area (Anrumai and Abu); the island of Bubaque, the most populated of the archipelago (Escadinhas, Bijante and Bruce); and in the islet of Adonga, inside Orango National Park (Adonga). Sites were sampled from the end of the wet season to the end of the dry season (October-April; Table 1), starting in January 2018 until April 2020 (months were not sampled consecutively within each year: S1 Table). This time of year was chosen as it overlaps with the presence of hundreds of thousands of migratory shorebirds in the archipelago, for which macroinvertebrates constitute a major part of their diet and are thus very likely to influence macrozoobenthos densities.

**Table 1:** Number of samples (sediment cores) collected in each site (n = 6) across months (n = 7), between 2018 and 2020, in the islands of Formosa, Bubaque and Orango, of the Bijagós Archipelago, Guinea-Bissau.

Period	Month	Island of Formosa		Island of Bubaque			Island of Orango	Total
		Anrumai	Abu	Bijante	Bruce	Escadinhas	Adonga	
End of wet season	October	12	0	12	11	12	57	104
	November	0	12	12	12	12	0	48
Early dry season	December	12	12	12	12	12	0	60
	January	8	7	12 + 12*	12	12 + 12*	0	51+24*
	February	16 + 12*	16 + 12*	16	15	16	20	99+24*
Late dry season	March	12	12 + 12*	16 + 12*	16	16 + 12*	31	103+36*
	April	12	12	12	12	12	0	60
**Total**		72 + 12*	71 + 24*	92 + 24*	90	92 + 24*	108	525+84*

* refers to cores collected in 2020, in which polychaetes were not sorted and were therefore excluded from diversity and community analysis.

Macrozoobenthos from four Classes (Polychaeta, Bivalvia, Gastropoda and Malacostraca) were collected using sediment cores of 0.00785 m^2^ of area for all sites except for Adonga, where cores had an area of 0.0113 m^2^, and max depth of 15 to 20 cm (often limited by rocks or shell banks). Sampling at all sites was conducted in areas where shorebirds where observed feeding. Except in one site (Adonga), polychaetes were not quantified in 2020, only bivalves, gastropods and malacostracans, as fieldwork came to a sudden halt due to the Covid-19 pandemic (Table 1, S1 Table). Core samples were divided in two fractions: the top 5 cm layer, where the majority of individuals are concentrated and therefore most of the harvestable biomass for intertidal predators, which was sieved through a 0.5 mm mesh size; and the bottom layer, which was sieved with a 1-mm mesh size. All individuals collected were stored in ethanol (70% or 96%), followed by later identification to the lowest taxonomic level possible with a magnifier lens, and different structures (see below) were measured to the closest mm. Identification was conducted with the support of a photographic reference collection built in this study (S1 Appendix), and also using identification keys for other areas published in books or journals [53–56]. Taxonomic names followed WoRMS (World Register of Marine Species; www.marinespecies.org).

### 2.3. Macrozoobenthos biomass estimation

A total of 26 taxa were considered for the biomass estimation, by including all taxa with densities above 0.8% of the total density of macrozoobenthos. The biomass of each individual found in almost all the core samples, was estimated from their size through taxa specific regression equations (Table 2). But for a small number of the collected cores (n = 20) the biomass of each individual was measured directly by following the methods described in Lourenço et al. (2017) [18], in which individuals were measured, dried to constant weight for 48h at 60°C and weighed (dry weight), and then incinerated in a muffle furnace for 2h at 500°C and weighted again (ash weight). The AFDM was calculated as the difference between dry weight and ash weight.

**Table 2:** Equations used to estimate biomass (AFDW, in mg) of different macroinvertebrate taxa, based on body size (BS, in mm).

(Sub) class	Taxa	AFDM equation (or average AFDM ± SD)	N individuals (N pools)	BS range (mm)	Source
Bivalvia	*Austromacoma nymphalis*	AFDM = 0.077*BS^1.902^	9	2–30	Lourenço et al. (2017) [18]
	*Diplodonta sp*.	AFDM = 0.002*BS^3.484^	47(1)	2–10	This study
	*Keletistes sp*.	AFDM = 0.091*BS^1.88^	56(26)	2–8	This study
	*Moerella distorta*	AFDM = 0.001*BS^3.64^	28(14)	3–12	This study
	*Pelecyora isocardia*	AFDM = 0.019*BS^2.646^	40	2–19	Lourenço et al. (2017) [18]
	*Senilia senilis*	AFDM = 0.035*BS^2.425^	16	2–26	Lourenço et al. (2017) [18]
	*Tagelus adansonii*	AFDM = 0.008*BS^2.52^	29	2–40	Lourenço et al. (2017) [18]
Polychaeta sedentaria	Capitelidae	AFDM = 0.537 ± 0.179	16(5)	8–23	This study
	Cirratulidae	AFDM = 0.14*BS^0.692^	16(5)	3–23	This study
	Maldanidae	AFDM = 0.009*BS^1.876^	16(10)	8–58	This study
	Orbiniidae	AFDM = 0.008*BS^1.675^	48(6)	8–43	This study
	Paraonidae	AFDM = 0.001*BS^2.325^	51(6)	8–28	This study
Polychaeta errantia	*Diopatra sp*.^*1*^	AFDM = 0.01*BS^1.517^	25	56–242	Lourenço et al. (2017) [18]
	*Glycera sp*.	AFDM = 0.009*BS^1.748^	17	9–51	Lourenço et al. (2017) [18]
	*Goniadopsis sp*.	AFDM = 0.513 ± 0.253	13(2)	8–13	This study
	Lumbrineridae	AFDM = 0.01*BS^1.422^	15	31–134	Lourenço et al. (2017) [18]
	*Marphysa sanguinea*	AFDM = 0.01*BS^1.517^	25	56–242	Lourenço et al. (2017) [18]
	Nereididae	AFDM = 0.009*BS^1.64^	15	8–45	Lourenço et al. (2017) [18]
	*Sigambra sp*.	AFDM = 0.19 ± 0.107	50(3)	8–13	This study
Malacostraca	*Afruca tangeri*	AFDM = 0.021*BS^3.222^	35	2–21	Lourenço et al. (2017) [18]
	Amphipoda	AFDM = 0.033*BS^1.37^	37(7)	3–8	This study
	Anthuridae	AFDM = 1.18 ± 0.33	6	6–8	Lourenço et al. (2017) [18]
	*Balsscalichirus balssi*	AFDM = 0.0003*BS^3.51^	22	9–27	Lourenço et al. (2017) [18]
	*Grandidierella sp*.	AFDM = 0.121 ± 0.045	28(3)	2–4	This study
Gastropoda	*Hyala sp*.	AFDM = 0.12 ± 0.04	40(2)	2–3	This study
	*Solariella sp*.	AFDM = 0.387 ± 0.143	29(3)	1–3	This study

Sample sizes (total number of individuals and, in some cases, in parenthesis is the number of pools of individuals), and size range are also indicated. Taxa for which significant regression equations were not obtained, an average AFDM value and SD is given. Averages and SD refer to the pools of individuals whenever it was necessary to pool individuals. ^1^ biomass was estimated using the equation for M. sanguinea, a closely related species from the same order (Eunicida), as no equation for this species was found or calculated in this study.

Previously published equations relating size to biomass for the study area were used whenever available [18], and for the remaining taxa new equations were calculated from individuals of different sizes sampled in the field, following the previously described procedure for obtaining the ADFM of each individual. For some species, a group of specimens had to be pooled together by size-class (Table 2), in order to obtain a detectable amount of biomass after incineration, and others were grouped into a pre-defined size class (e.g., size between 10 and 15mm), for which the body size attributed to the individual was the average value of that size class (in this case 12.5mm). New regression equations were calculated by determining the nonlinear least-squares estimates of the parameters of the model AFDM = a*BS^b^ [57–59] where BS is the Body Size in mm (antero-posterior length in bivalves and gastropods, total length in polychaetes and most malacostracans, and carapace width in crabs), using the *nls* function in R Software version 3.5.0 [60]. Whenever significant regression equations were not obtained for a certain taxon (i.e. size variation had no effect on biomass, in some cases potently due to limited sample size), an average AFDM value was used (regardless of the individual’s size) obtained from previously published studies or from incinerated individuals (Table 2).

### 2.4. Data analysis

Temporal variation was analysed using two variables: 1) month, a continuous variable that consisted of a pool of the samples of each sampled month in any year; and 2) period, a categorical variable that consisted in aggregating months into three main periods: end of wet season (October and November), early dry season (December, January and February) and late dry season (March and April; Table 1). Month was used for the univariate analysis and period for the multivariate analysis (see below). Aggregation into periods served the main purpose of increasing the sample size for the community analysis, allowing a more robust application of multivariate statistics. Those periods were selected because they represent three different environmental moments: the transitional period just after the rainy season (end of wet), with a stronger prevalence of the influence from rainfall (especially continental runoff) during the preceding months, which may promote inter-seasonal differences in the macrozoobenthic community [61, 62]; and the first (early dry) and last (late dry) months of the dry season, with the climate becoming increasingly drier and hotter. Furthermore, these three periods also represent three different moments in relation to the predation pressure exerted by migratory shorebirds on macrozoobenthos: the end of wet season, corresponding to the progressive arrival of shorebirds [37, 63] and thus relatively lower predation pressure on macrozoobenthos; the early dry season, corresponding to the period when shorebirds forage only to meet their daily energy requirements; and the late dry season, when shorebirds typically increase their daily intake in preparation for the migration towards the breeding areas [64–66]. In order to check that pooling of months of different years was acceptable, we tested for differences in density on samples collected in the same month (and site) in different years, using two-sample Wilcoxon tests. This showed that in the large majority (21 out 26 comparisons) of cases, there were no significant differences in the densities of macrozoobenthos between years (S2 Table).

In order to describe the spatial and temporal variation in overall density (ind.m^-2^) and biomass (mg of AFDM.m^-2^) of macrozoobenthos, and to assess if temporal patterns varied between sites, we conducted a Generalised Linear Model with negative binomial family (GLMnb), to account for overdispersion, and used the log link function. To model the variation in density, we used the number of individuals (all macrozoobenthos) counted in each core as the dependent variable, and to model variation in the biomass we used mg of AFDM in each core. The log of the area of each sampling core was used as an offset in all models, as this varied between sites, and the explanatory variables were site (with six levels) and month (continuous variable, coded sequentially from 1 to 7, starting in October). We also explored the interaction between the two explanatory variables. Model selection was undertaken using Akaike Information Criterion (AIC), by fitting all possible model combinations and selecting the model with the lowest AIC. When AIC differ less than two, a common “rule of thumb”, indicating no support for differences between competing models (see [67]), we selected the most parsimonious model. The same approach was used to describe the variation in spatial and temporal patterns for each of the major macrozoobenthic (sub)classes (Bivalvia, Polychaeta sedentaria, Polychaeta errantia, Gastropoda and Malacostraca). When differences between sites were significant, we performed post-hoc Tukey HSD pairwise contrasts to determine which sites were different from each other. When the interaction between site and month was significant for a given macrozoobenthos (sub)class, we computed individual GLMnb within each site to assess the significance of the variation of density and biomass along months.

We computed Shannon-Wiener diversity index and species richness for each site and overall to compare with previous studies in the Bijagós Archipelago and with macrozoobenthos communities in other tropical and temperate intertidal systems elsewhere.

In order to explore spatial variation in the community composition (i.e. the identity and relative abundances of all taxa in the community) and assess if temporal variation is similar between sites, we first calculated zero-adjusted Bray-Curtis dissimilarities [68], which summarizes differences between densities of taxa (ind.m^-2^) among samples (cores) with many zeros, using a log (x+1) transformation to reduce the weight of the most abundant species. We then used this dissimilarity matrix as the dependent variable on a permutational analysis of variance (PERMANOVA), with site (six levels) and period (three levels) as explanatory variables. We also tested the interaction between site and period and when the interaction was significant, we further inspected differences among sites separately for each level of period using PERMANOVAs, having site as the explanatory variable. Post-hoc PERMANOVAs with Bonferroni’s p-value adjustment for multiple comparisons were used to assess significant differences between sites. PERMANOVA’s assumption of homogeneity of multivariate dispersal was tested using *betadisper* and *permutest* functions of the VEGAN package, which simultaneously tested differences in community diversity between sites. In the event of heterogeneity of multivariate dispersal, PERMANOVA remains a robust test in the presence of balanced designs [69]. Nonetheless, in these cases we regarded the results of PERMANOVA with care, as the reasons for the differences may not be straightforward in some cases.

To visualize the differences in the centroids and in multivariate dispersal of the community composition among sites for each period, we computed Canonical Analysis of Principal Coordinates based on discriminant analysis (with three axis; hereafter referred to as CAP), using the function *CAPdiscrim*, from the BiodiverityR package [70] following the methods described in Anderson and Willis (2003) [71]. We used zero-adjusted Bray-Curtis dissimilarity matrix (based on densities) and the variable site for the constrained ordinations. Finally, to assess which species best characterized the differences in the community composition, we performed multiple linear regressions with a permutational test of significance of the fitted vectors, between the densities of each species (with a log (x+1) transformation) and the axis of the CAP ordination. For clarity, only the species with significant fits and R^2^≥ 0.15 were plotted as vector arrows over the CAP ordinations, with arrow length proportional to R^2^ value of the regression, thus informing on the importance of each species in the macrozoobenthic community. Plots showing all the species with significant fits, regardless of their R^2^, were also performed and presented in the Supporting Information. All the analysis were performed in R Software version 3.5.0 [60] and results are shown as mean ± SE, unless otherwise stated.

### 2.5. Inclusivity in global research

Permits to access field sites and sample were given by Instituto da Biodiversidade e das Áreas Protegidas Dr. Alfredo Simão da Silva, under the guardianship of the Ministry of Environment. Additional information regarding the ethical, cultural, and scientific considerations specific to inclusivity in global research is included in the Supporting Information (S1 Checklist).

## 3. Results

### 3.1. Spatial and temporal variation in density and biomass of macrozoobenthos

**Fig 2:**
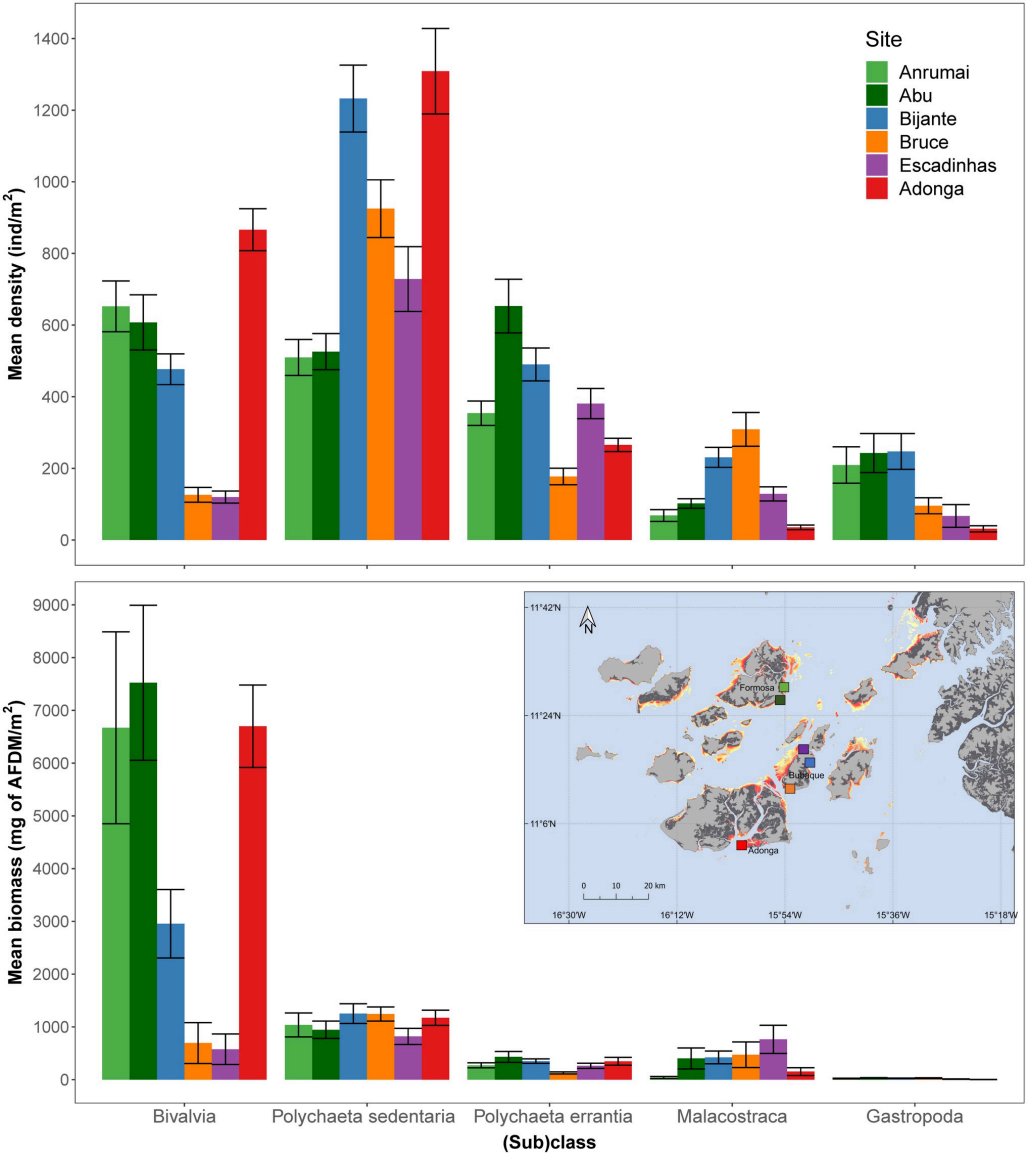
Variation in the mean density (ind.m^-2^) and biomass (mg AFDM.m^-2^), with standard error bars, of major macrozoobenthos (sub)classes sampled in each site. Map of the Bijagós archipelago (inset) is shown, with indication of the spatial distribution of the study sites, represented by coloured squares with matching colour scheme.

From a total of 9907 individuals sampled in 609 sediment cores, the Bijagós intertidal flats had an average macrozoobenthos density of 1871 ± 58.3 ind.m^-2^, corresponding to a mean biomass of 5.65 ± 0.41 gAFDM.m^-2^ (Table 1; S3 Table). Sedentary polychaetes were the most abundant (sub)class, representing 41% of total density, followed by bivalves (24%), errant polychaetes (17%), malacostracans (8%) and gastropods (8%). Regarding biomass, bivalves were the predominant class, representing 72% of the total biomass, followed by sedentary polychaetes (17%), malacostracans (7%), errant polychaetes (4%) and lastly gastropods, which represented less than 1% of the total (Fig 2). When aggregating all months, mean densities and biomass varied markedly across sites for each macrozoobenthic (sub)class (Fig 2).

#### 3.1.1. Variation in macrozoobenthos density

Overall density of macrozoobenthos varied significantly among sites (p<0.001), with Adonga having the highest mean density, significantly higher than all sites except Bijante, and followed by Abu, Anrumai, Bruce and Escadinhas, the later having significantly lower densities than all other sites (Table 3; S3 and S4 Tables). Overall density increased significantly throughout months (p<0.001; Table 3), and the interaction between month and site was not significant (interaction term, p = 0.07). Removing Adonga, the site with only three months sampled, from the GLM analysis presented in Table 3, yielded the same overall results.

**Table 3:** Results of Generalized linear models (GLM) with negative binomial family, describing temporal and spatial variation on density (individuals per m^2^) and biomass (mg AFDM.m^-2^) of macrozoobenthos of major (sub)classes, using core size as an offset.

	Density (individuals per m^2^)				Biomass (mg AFDM.m^-2^)			
	Estimate	Std. Error	z value	Pr(>|z|)	Estimate	Std. Error	z value	Pr(>|z|)
**Overall**								
Anrumai	7.190	0.10	-19.5	**<0.001**	8.970	0.19	-1.3	0.191
Abu	0.081	0.11	0.8	0.453	0.135	0.19	0.7	0.483
Bijante	0.333	0.10	3.3	**0.001**	-0.519	0.18	-2.8	**0.005**
Bruce	-0.020	0.11	-0.2	0.856	-1.119	0.19	-5.8	**<0.001**
Escadinhas	-0.337	0.10	-3.2	**0.001**	-1.269	0.18	-6.9	**<0.001**
Adonga	0.479	0.10	4.6	**<0.001**	0.064	0.19	0.3	0.734
Month	0.051	0.02	3.4	**0.001**	0.0001	0.03	0.0	0.997
**Bivalvia**								
Anrumai	5.990	0.14	-22.5	**<0.001**	9.105	0.30	-0.3	0.729
Abu	-0.121	0.14	-0.9	0.393	0.151	0.32	0.5	0.633
Bijante	-0.304	0.14	-2.2	**0.027**	-0.803	0.30	-2.7	**0.008**
Bruce	-1.636	0.17	-9.5	**<0.001**	-2.357	0.32	-7.4	**<0.001**
Escadinhas	-1.677	0.16	-10.5	**<0.001**	-2.501	0.30	-8.2	**<0.001**
Adonga	0.440	0.14	3.2	**0.001**	-0.117	0.31	-0.4	0.706
Month	0.106	0.02	4.9	**<0.001**	-0.064	0.04	-1.4	0.15
**Polychaeta sedentaria**								
Anrumai	6.380	0.25	-11.2	**<0.001**	7.067	0.36	-6.0	**<0.001**
Abu	0.192	0.39	0.5	0.62	-1.018	0.56	-1.8	0.067
Bijante	0.709	0.32	2.2	**0.026**	-0.572	0.47	-1.2	0.223
Bruce	0.088	0.32	0.3	0.786	-0.221	0.47	-0.5	0.635
Escadinhas	-0.570	0.33	-1.7	0.087	-0.323	0.47	-0.7	0.491
Adonga	0.881	0.28	3.1	**0.002**	0.267	0.41	0.7	0.513
Month	-0.034	0.05	-0.6	0.523	-0.029	0.07	-0.4	0.701
Abu:month	-0.035	0.08	-0.4	0.664	0.196	0.11	1.7	0.086
Bijante:month	0.041	0.07	0.6	0.55	0.172	0.10	1.7	0.083
Bruce:month	0.118	0.07	1.7	0.086	0.094	0.10	1.0	0.342
Escadinhas:month	0.209	0.07	3.0	**0.003**	0.020	0.10	0.2	0.842
Adonga:month	0.007	0.06	0.1	0.912	-0.063	0.09	-0.7	0.484
**Polychaeta errantia**								
Anrumai	5.920	0.26	-12.7	**<0.001**	6.250	0.37	-7.9	**<0.001**
Abu	0.614	0.38	1.6	0.104	0.415	0.57	0.7	0.464
Bijante	-0.565	0.34	-1.6	0.101	-0.917	0.50	-1.8	0.064
Bruce	-1.288	0.39	-3.3	**0.001**	-1.650	0.53	-3.1	**0.002**
Escadinhas	-1.166	0.36	-3.2	**0.001**	-1.213	0.51	-2.4	0.016
Adonga	-0.385	0.30	-1.3	0.197	-0.829	0.43	-1.9	0.053
Month	-0.011	0.05	-0.2	0.835	-0.158	0.08	-2.0	0.049
Abu:month	0.0003	0.08	0.0	0.997	0.021	0.12	0.2	0.862
Bijante:month	0.199	0.07	2.8	**0.005**	0.279	0.11	2.6	**0.009**
Bruce:month	0.137	0.08	1.7	0.089	0.216	0.11	1.9	0.059
Escainhas:month	0.273	0.07	3.7	**<0.001**	0.280	0.11	2.6	**0.01**
Adonga:month	0.026	0.07	0.4	0.688	0.282	0.10	3.0	**0.003**
**Malacostraca**								
Anrumai	4.816	0.46	-9.6	**<0.001**	5.670	0.80	-4.4	**<0.001**
Abu	-0.363	0.69	-0.5	0.601	-2.974	1.21	-2.5	**0.014**
Bijante	-0.231	0.57	-0.4	0.683	0.587	0.99	0.6	0.553
Bruce	0.217	0.56	0.4	0.698	-1.285	1.01	-1.3	0.205
Escadinhas	0.172	0.57	0.3	0.763	1.159	0.99	1.2	0.24
Adonga	-1.058	0.55	-1.9	0.053	-2.592	0.93	-2.8	**0.005**
Month	-0.139	0.10	-1.4	0.162	-0.524	0.19	-2.8	**0.006**
Abu:month	0.175	0.14	1.2	0.222	1.119	0.26	4.3	**<0.001**
Bijante:month	0.326	0.12	2.7	**0.007**	0.473	0.23	2.1	**0.036**
Bruce:month	0.298	0.12	2.5	**0.013**	0.874	0.23	3.8	**<0.001**
Escadinhas:month	0.108	0.12	0.9	0.381	0.478	0.23	2.1	**0.034**
Adonga:month	0.074	0.13	0.6	0.563	1.008	0.22	4.6	**<0.001**
**Gastropoda**								
Anrumai	4.375	0.64	-7.6	**<0.001**	1.764	0.45	-16.4	**<0.001**
Abu	0.771	0.95	0.8	0.416	0.342	0.36	1.0	0.338
Bijante	0.358	0.82	0.4	0.66	0.245	0.36	0.7	0.49
Bruce	-1.610	0.92	-1.8	0.079	0.327	0.37	0.9	0.377
Escadinhas	0.300	0.84	0.4	0.722	-0.725	0.44	-1.6	0.101
Adonga	-3.125	0.94	-3.3	**0.001**	-2.162	0.72	-3.0	**0.003**
Month	0.197	0.13	1.5	0.127	0.266	0.07	3.9	**<0.001**
Abu:month	-0.126	0.19	-0.7	0.505				
Bijante:month	-0.028	0.17	-0.2	0.866				
Bruce:month	0.189	0.19	1.0	0.309				
Escadinhas:month	-0.315	0.18	-1.8	0.076				
Adonga:month	0.299	0.19	1.6	0.117				

The site Anrumai was used as reference. Significant results are marked in bolt. Regressions results of biomass:month and density:month in Adonga were not considered because only three months were sampled in that site.

At the (sub)class level, Adonga had highest mean density of bivalves and sedentary polychaetes (the later together with Bijante), but lowest of gastropods and malacostracans (S3 and S4 Tables). Bruce had the highest densities of malacostracans, but the lowest of bivalves (together with Escadinhas) and errant polychaetes (S3 and S4 Tables). This polychaete subclass had its highest density in Abu, where gastropods were also found to be the highest (together with Bijante), while Abu had the lowest densities of sedentary polychaetes, together with Anrumai (S3 and S4 Tables).

**Fig 3:**
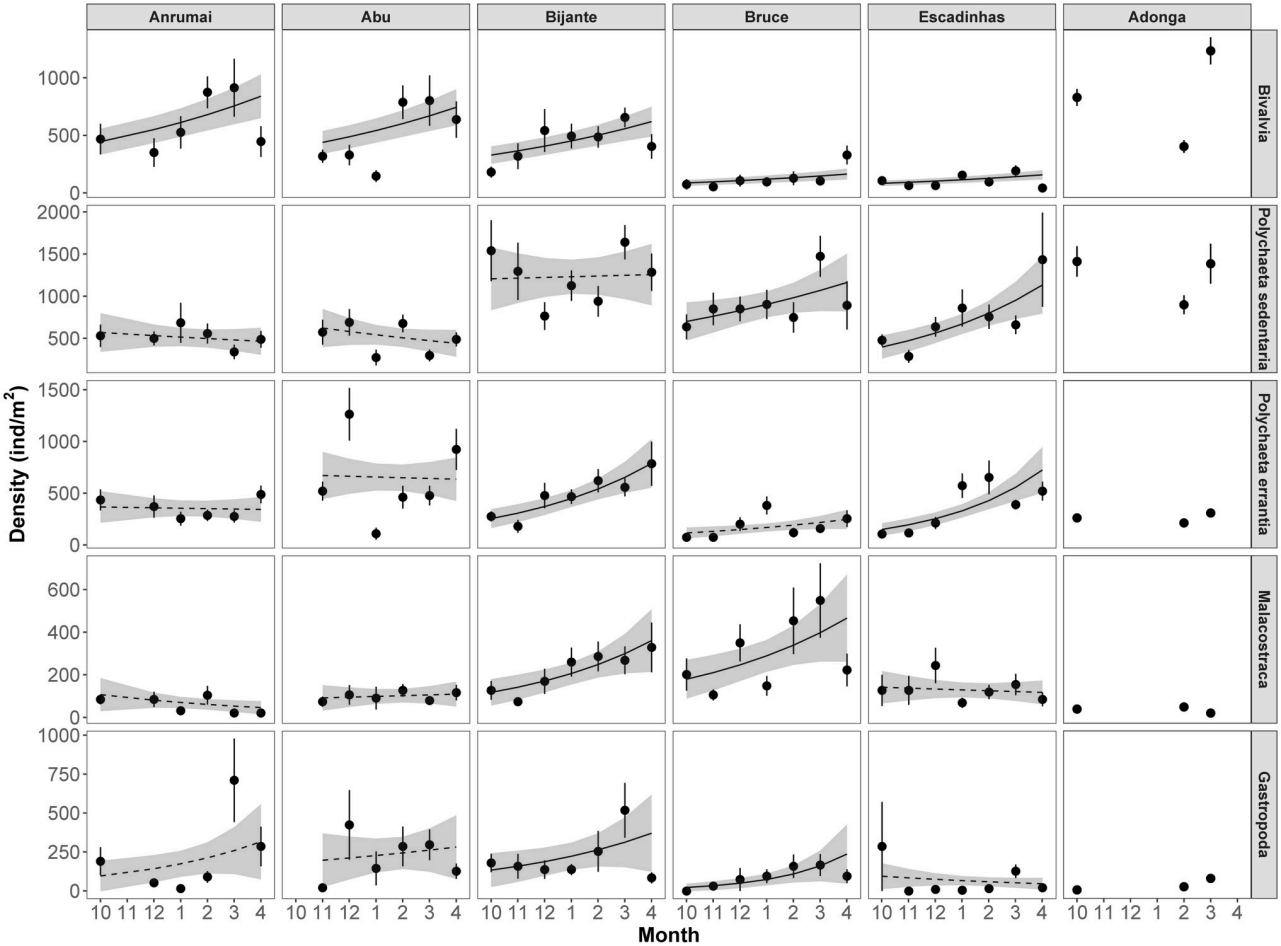
Temporal (monthly) variation in the density (individuals per m2) of major macrozoobenthic invertebrate (sub)classes (rows; note varying scales on y axis) in each site (columns) throughout months. Black circles and vertical lines represent mean with associated standard errors. Black lines represent Generalized linear models (family negative binomial, with log link) fitted to data points, with grey areas showing 95% confidence intervals. Significant relationships are represented by solid lines, and non-significant by dashed lines. Estimates for the variation of density for each unit of month and p-values are shown in each panel. Bivalves are the only (sub)class for which no significant interaction was found between site and month (Table 3), and therefore has only one estimate of month and p-value for all sites. Regressions lines for Adonga were not computed due to only having 3 sampled months.

Bivalve density increased significantly across months regardless of site (Table 3, Fig 3). Sedentary and errant polychaetes also increased significantly in Escadinhas (Fig 3) with the former showing the same pattern on Bruce and the latter on Bijante. Malacostracans and gastropods increased in Bruce and Bijante, with the later also increasing in Adonga (Fig 3).

#### 3.1.2. Variation in macrozoobenthos biomass

The biomass of macrozoobenthos varied significantly among sites (p<0.001), with Abu, Adonga, and Anrumai having significantly higher biomass than all other sites, followed by Bijante, with Bruce and Escadinhas having significantly less biomass than the remaining sites (Table 3; S3 and S4 Tables). Overall, biomass did not vary significantly throughout months (p = 0.997), and the interaction term was also not significant (interaction term, p = 0.16), even when removing Adonga from the analysis.

At the (sub)class level, Abu, Adonga and Anrumai had the highest biomass of bivalves, with the last two sites having the lowest biomass values of gastropod and malacostracans, respectively (S3 and S4 Tables). The highest biomass of malacostracans was found in Escadinhas and of errant polychaete in Abu (S3 and S4 Tables).

**Fig 4:**
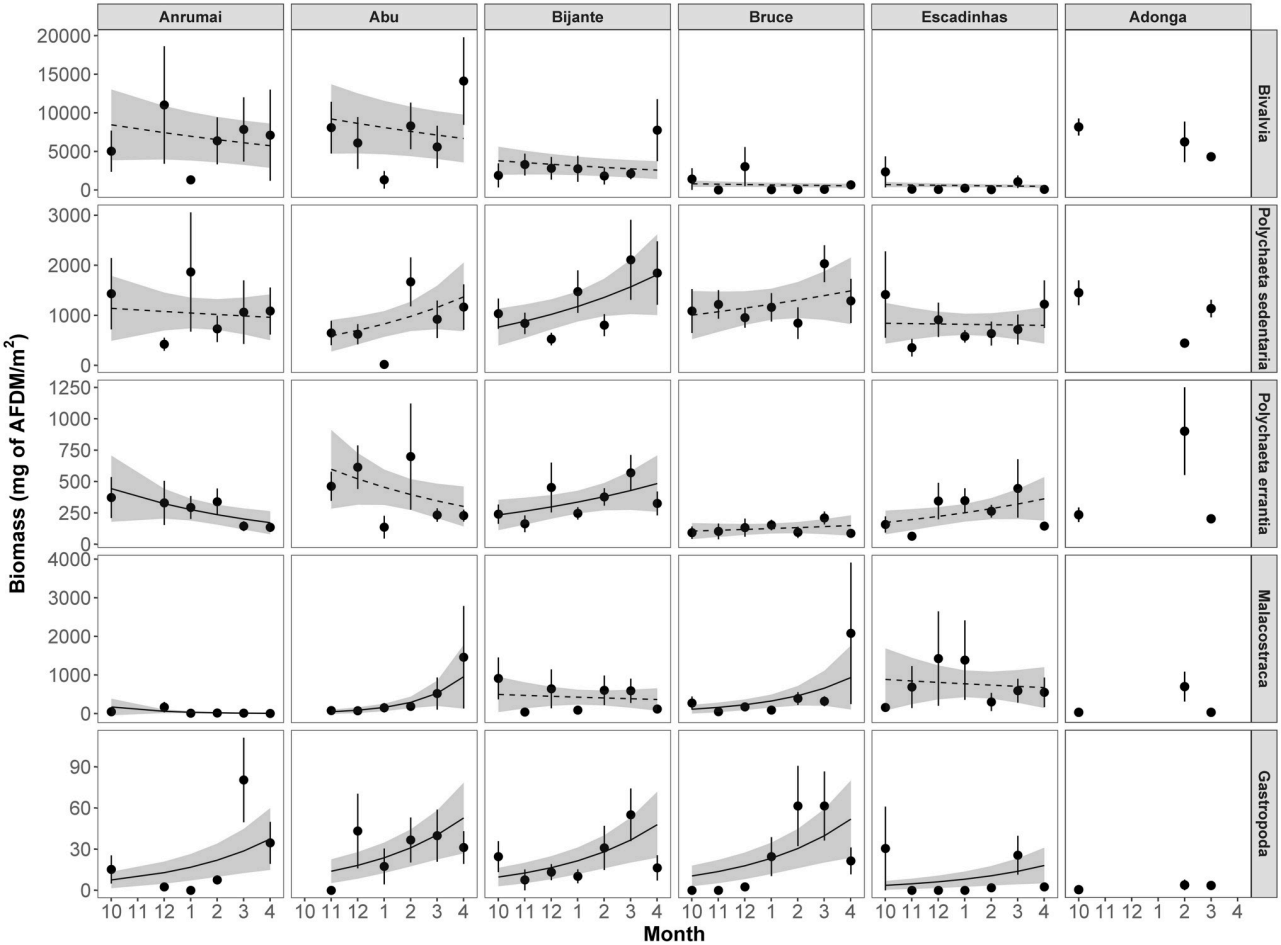
Temporal (monthly) variation in the biomass (mg of AFDM.m^-2^) of major macrozoobenthic invertebrate (sub)classes (rows; note varying scales on y axis) in each site (columns) throughout months. Black circles and vertical lines represent mean and standard errors, respectively. Black lines represent Generalized linear models (family negative binomial, with log link) fitted to data points, with grey areas showing 95% confidence intervals. Significant relationships are represented by solid lines, and non-significant by dashed lines. Estimates for the variation of density for each unit of month and p-values are shown in each panel. Bivalves and Gastropods are the only (sub)classes for which no significant interaction was found between site and month (Table 3), and therefore has only one estimate of month and p-value for all sites. Regressions lines for Adonga were not computed due to only having 3 sampled months.

Bivalve biomass did not vary across months, whereas the remaining (sub)classes showed different patterns of monthly variation, the majority of which were positive (Table 3, Fig 4). Both sedentary and errant polychaete biomass increased significantly in Bijante, but decreased in Adonga (sedentary) and Anrumai (errantia; Table 3, Fig 4). The later site also showed a decrease in malacostracan biomass, which in turn increased in three other sites (Abu, Bruce and Adonga). Gastropod was the only class with an increase in biomass regardless of sites (Table 3, Fig 4).

### 3.2. Variation in macrozoobenthos community composition and diversity

Overall, at least 88 taxa were identified in all sites, at different taxonomical levels (S3 Table). To assess the diversity, species richness and community composition (analysed based on the density of each species), these 88 taxa were aggregated into 82 to exclude taxa repetitions along different taxonomical levels (e.g. when some individuals of a given family were possible to identify at the species level, but most were not, all were aggregated at the family level). Overall, we found a Shannon-Wiener of 3.3 when aggregating samples from all sites. Site specific species richness and diversity are presented in Supporting Information (Table 1 in S2 Appendix).

#### 3.2.1. Overall spatial and temporal differences

**Fig 5:**
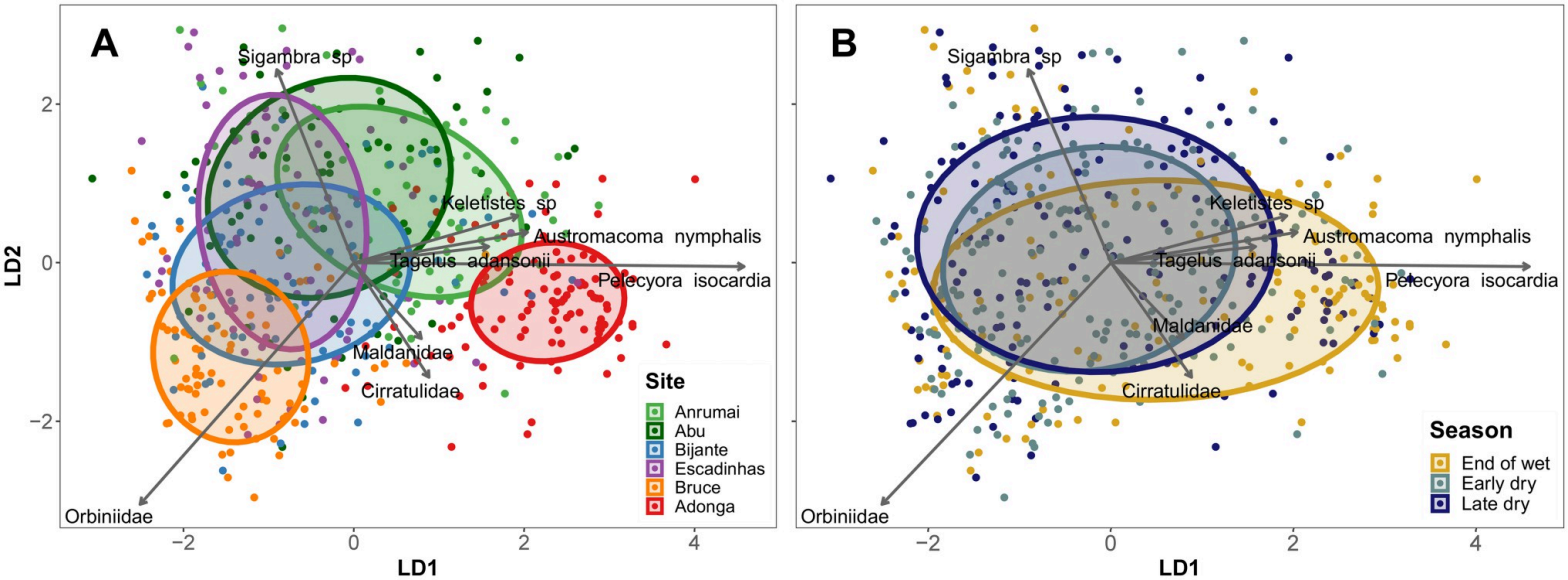
Canonical analysis of Principal Components based on discriminant analysis, grouping the data points (cores) per site (A) and period (B) in a dual-axis linear discriminant space. Ellipses represent 60% of the data for each group. Arrows show species with the most influence in the distribution of data points, and the size of the arrows are scaled according to the R^2^ of each regression between the densities of each species and the axis of the CAP ordination (i.e., longer arrows represent more influent species). Species with significant fits but with an R^2^ smaller than 0.15 are omitted.

The macrozoobenthic community composition showed overall significant differences among sites (PERMANOVA F_5,507_ = 22.76, R^2^ = 0.17, p<0.001) and periods (but with a small amount of explained variance; PERMANOVA F_2,507_ = 6.4, R^2^ = 0.02, p<0.001), and the variation between periods was significantly different among the sites (PERMANOVA interaction term, F_10,507_ = 2.6, R^2^ = 0.04, p<0.001). CAP ordinations of sites suggest that Adonga has the most different community composition, mostly due to the high densities of the bivalves *P*. *isocardia*, *A*. *nymphalis*, *Keletistes* sp. and *T*. *adansonii* (Fig 5A). The errant polychaete *Sigambra* sp. appeared to be especially abundant in Abu and Escadinhas, while a higher density of the sedentary polychaete Orbiniidae seemed to be responsible for the community composition differences in Bruce. Multivariate dispersions (community diversity) were significantly different across the sites (Permutation test, F_5,519_ = 20.2, p = 0.001), with Adonga as the only site with significantly smaller dispersion than all the others (S5 Table). Temporal patterns are unclear in the overall CAP ordinations and difficult to interpret at such wide level (Fig 5B). Furthermore, there were no significant differences in multivariate dispersions between periods (Permutation test, F_2,522_ = 2.57, p = 0.087).

#### 3.2.2. Differences between sites in each period

When comparing the community composition in each period, there were significant differences between sites in all periods (PERMANOVAs, end of wet season: F_5,146_ = 10.71, R^2^ = 0.27, p = 0.001; early dry season: F_5,204_ = 6.32, R^2^ = 0.13, p = 0.001; late dry season: F_5,157_ = 11.48, R^2^ = 0.27, p = 0.001). The same holds for differences between sites in the multivariate dispersion (permutation tests, end of wet season: F_5,146_ = 8.85, p = 0.001; early dry season: F_5,204_ = 8.00, p = 0.001; late dry season: F_5,157_ = 8.41, p = 0.001).

**Fig 6:**
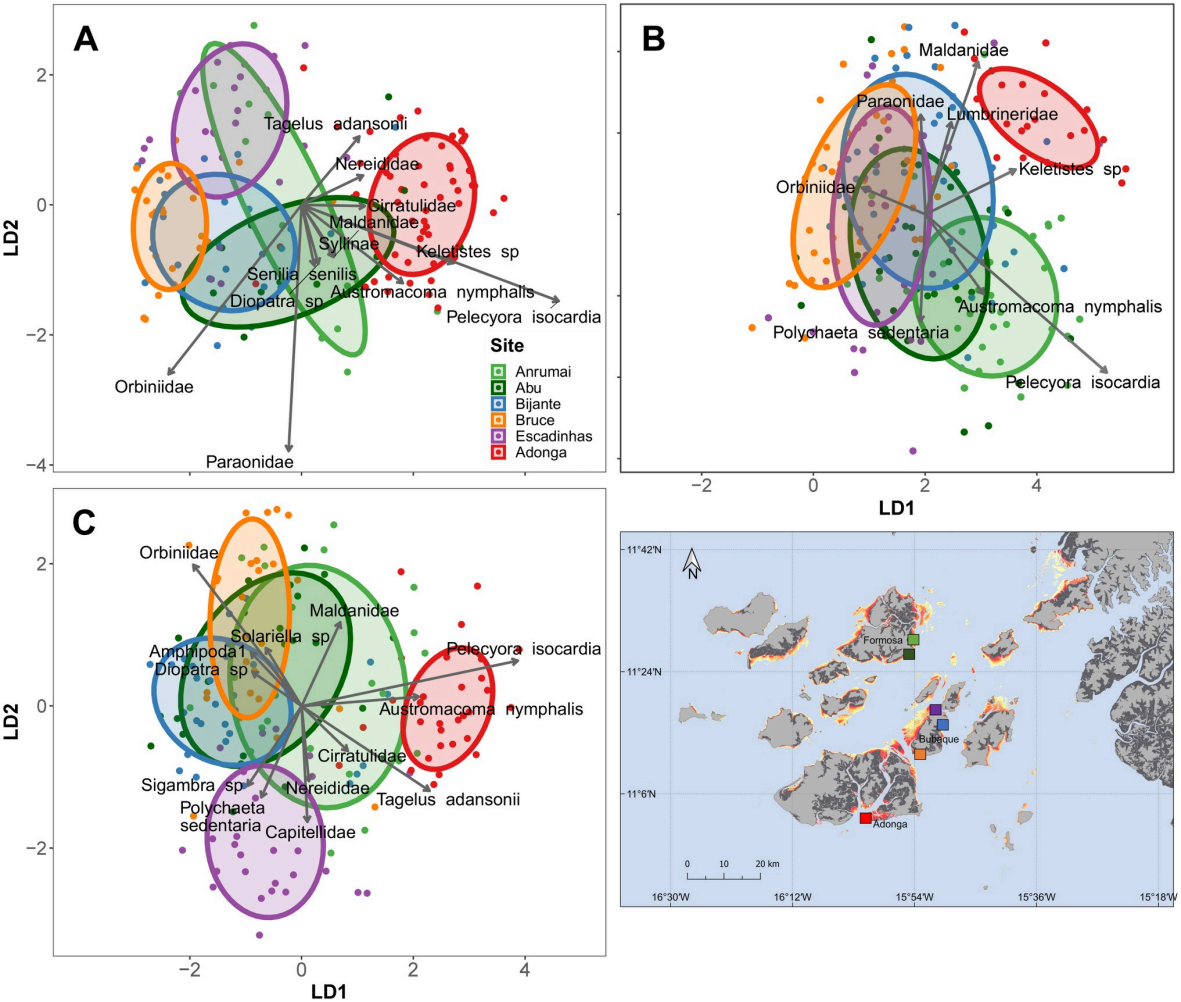
Canonical analysis of Principal Components based on discriminant analysis (made with 3 axis), grouping the data points (cores) per site, in the end of wet season (A: October–November); early dry season (B: December, January and February); and end late dry season (C: March and April,) in a dual-axis linear discriminant space. Ellipses represent 60% of the data for each group. Arrows show species with the most influence in the distribution of data points, and the size of the arrows is scaled according to the R^2^ of each regression (i.e., longer arrows represent more influential species). Species with a significant fit but with an R^2^ smaller than 0.15 are omitted. A map of the Bijagós Archipelago is shown, with indications of the study site locations represented as squares with a matching colour scheme.

In the end of the wet season (October and November) the most similar communities were those in Anrumai and Abu (S6 Table). Adonga was the site with the most different community composition, influenced by the importance of the bivalves *P*. *isocardia*, *Keletistes sp*. and *A*. *nymphalis*. In Abu the sedentary polychaetes Paraonidae and Orbiniidae were important families to differentiate this community from the others, while the Orbiniidae were also important in Bruce and Bijante (Fig 6A). The significant differences in multivariate dispersal at the end of wet period were mostly due to Bruce and Adonga having significantly smaller dispersion in their communities in comparison to the other sites (S5 Table; Fig 6A).

In early dry season (December, January and February), the communities in Adonga remained the most distinct (S6 Table, Fig 6B). In this period, however, it is noticeable that the sedentary polychaete Maldanidae replaced the bivalves that dominated the community composition of Adonga in the previous period, especially *P*. *isocardia* (Fig 6B). This bivalve was abundant in this period in Anrumai instead, increasing markedly its importance at this site, together with *A*. *nymphalis*, although to a lesser extent. In Bruce, Orbiniidae maintained their importance in comparison to the previous period, while increasing in Escadinhas. In the early dry season, the multivariate dispersal of the macrozoobenthic community in Adonga was also significantly smaller than in all other sites, in which dispersal increased instead (S5 Table, Fig 6B).

Finally, in late dry season, most sites remain significantly different from each other, except for Anrumai and Abu, as in the end of wet season. Adonga also remained the most different site (S6 Table, Fig 6C), and similarly to the end of wet season, is strongly influenced by the bivalves *P*. *isocardia* and *A*. *nymphalis*, which increased markedly from early dry season to late dry season, as well as the bivalve *T*. *adansonii*, that became important in Adonga only in late dry season. In Bruce, Orbiniidae maintained its importance from early to late dry season, while in Bijante Orbiniidae’s importance increased to a similar importance as at the end of the wet season. The community in Escadinhas shifted to be dominated by sedentary polychaetes (including Capitellidae) and the errant polychaete *Sigambra* sp. The multivariate dispersion also differs significantly, mostly because of the significantly higher dispersion in Anrumai and Abu in this final period (S5 Table).

## 4. Discussion

In this study we undertook a large sampling effort over the intertidal flats of the Bijagós Archipelago, covering six sites and seven months (n = 609 cores), to describe the spatial and temporal variation of macrozoobenthos density, biomass, and community diversity and composition. We found a highly diverse community, with more than 88 taxa, but with typically modest densities and biomass. With both univariate and multivariate approaches, we showed significant differences in the macrozoobenthos density and community structure among sites. Interestingly, overall density increased linearly throughout months, with all significant relationships being positive across sites and (sub)classes. However, contrarily to the expected reduction in biomass due to shorebird predation in this period, we found no clear overall trends for biomass when aggregating all sites, and at the site level most of the significant relationships across sub(classes) were positive (although two were not). These patterns dispute the expected depletion in the macrozoobenthos community by the end of the dry season due to intense predation pressure from migratory shorebirds, which adds to the local fish predation over the studied period. The findings are at odds with the observation at Banc d’Arguin, Mauritania, where depletions over the shorebird predation season did seem to occur [29].

### 4.1. Overall patterns of macrozoobenthos communities

On average, macrozoobenthos of the Bijagós Archipelago had a density of 1871 ± **58** ind.m^-2^, corresponding to an average biomass of 5.65 ± 0.41 gAFDM.m^-2^. These values are in accordance with previous studies done locally at smaller spatial and temporal frames [46, 72]. But while density values are within the expected range for tropical sites [20], our biomass values were overall much lower when compared to the nearby intertidal area of the Banc d’Arguin, which wielded 17.0 gAFDM.m^-2^ [57]. Even larger differences are observed when compared to biomass values reported for temperate intertidal areas within the East Atlantic Flyway, as the Tagus estuary, or the Wadden Sea (with 21.9 g of Dry Mass.m^-2^ and 26.6 gAFDM.m^-2^ respectively) [73, 74].

We found that sedentary polychaetes, the most abundant taxonomic group (41% of total density), accounted for only 17% of the total macrozoobenthos biomass, whereas the second most common group, bivalves (27% of total density), had a disproportionally high contribution towards biomass, representing 72% of the total. This pattern of bivalves occurring at low densities but high biomass is typical in other intertidal systems, both temperate, such as the Tagus estuary where the bivalve *Scrobicularia plana* is responsible for most of the biomass [75, 76], and tropical, like in South Sumatra in Indonesia where the Arcidae bivalve *Anadara granosa* takes that role [77]. In our study, a large proportion of bivalve biomass (46%) is composed of individuals of *S*. *senilis* (Arcidae family), which is in line with previous studies in the Bijagós Archipelago [45, 46] and is similar to the assemblages in Banc d’Arguin [29, 78]. This species is important for example in the diet of Oystercatchers [79] and some specialized rays [80], and although it is not consumed by most shorebirds [18], on some occasions it can also be part of the diet of some of these species, like for Whimbrel [81], while also being commercially harvested and one of the major protein sources for local people [82].

Tropical intertidal flats are known to often show higher macrozoobenthos diversity than their temperate counterparts [11, 20, 33], and the Bijagós Archipelago is no exception. Overall, we found that species richness (88 taxa) and diversity (Shannon-Wiener index of 3.3) were considerably higher than previously reported values for this area. Lourenço et al. (2018) [46] reported a species richness of 40 taxa with a Shannon-Wiener index of 2.89, whilst Meijer et al. (2021) [45] found 48 taxa. These differences are likely due to the more restricted sampling presented in those studies, which were also more constrained in time and/or space, even though in both studies all sites sampled in the present study were included. It should nevertheless be noted that the accumulation curves produced in our study (Fig 1 of S2 Appendix) show an overall rapid increase in the number of taxa found for each additional core and despite our larger sampling effort none has reached the asymptote. This suggests that further sampling may render even higher values of diversity. Furthermore, species richness in another intertidal system in the region, the Banc d’Arguin, in Mauritania, is reported to be higher (111 taxa) [83] despite intrinsic differences between systems, it could be expected that more species may be found in the Bijagós, particularly in a sampling scheme that covers other periods of the annual cycle.

The macrozoobenthic community composition was shown to be overall well segregated in the CAP space, with the community structure gradients particularly dominated by the differences in the occurrence and density between eight key taxa. The first axis of the CAP ordination showed mainly the distribution of sampling cores along the gradients of abundance of the bivalve species, while the second axis mainly reflected the gradient along the abundance of the polychaete taxa. This suggests a diverse community that depends on several important taxa, an important trait of stable and resilient ecosystems [84, 85]. These results partially agree with those reported by Lourenço et al. (2018) [46], in which also several (ca. 9) polychaete worms and bivalves were statistically explaining the community structure gradients. However, a notable difference is the presence of the fiddler crab *Afruca tangeri* as a key species in Lourenço et al. (2018) [46], which was not the case in our work. This discrepancy is likely related to methodological differences: we sampled macrozoobenthos using exclusively core samplers (which tend to miss crabs for being considerably mobile) whereas Lourenço et al. (2018) [46] conducted a separate method specific for sampling fiddler crabs. This likely resulted in an underestimation of their importance in our study. Nevertheless, fiddler crabs are ecosystem engineers within the intertidal flats of the Bijagós Archipelago, and while not being direct predators of macrozoobenthos [86], they might negatively impact macrozoobenthos abundance and structure the community composition of both benthic invertebrates and shorebird consumers [28]. Thus, their inclusion within the lot of key species is warranted, as previously suggested by Lourenço et al. (2018) [46].

### 4.2. Spatial variation of macrozoobenthos communities

We showed overall significant spatial variation in both density and biomass of macrozoobenthos when comparing sites, and this was also true at the (sub)class level for the majority of macrozoobenthos. Considering density, when aggregating all macrozoobenthos (sub)classes, four sites stand out, Adonga and Bijante with comparatively high mean densities (2507 ± 157 ind.m^-2^ and 2321 ± 149 ind.m^-2^, respectively), and Escadinhas and Bruce with lower densities (1195 ± 100 in.m^-2^ and 1633 ± 119 in.m^-2^, respectively), than all other sites. These patterns were the same for Adonga, Escadinhas and Bruce when considering mean biomass (8.37 ± 0.83 g AFDM m^-2^, 2.21 ± 0.4 g AFDM m^-2^ and 2.57 ± 0.47 g AFDM m^-2^, respectively), but the high density found in Bijante did not translate into high mean biomass (4.68 ± 0.67 g AFDM m^-2^). This is likely due to the comparatively lower prevalence of bivalves in this site (which represented the main source of biomass in the whole system), in comparison to other sites like Anrumai, Abu and Adonga, and which in Bijante were replaced by higher density of sedentary polychaetes. Additionally, the mean size of bivalves in Bijante was the lowest of all sites (5.0 ± 0.2 cm in Bijante vs 7.6 ± 0.3 cm in Adonga and 5.5 cm for both Anrumai and Abu, with SE of 0.4 and 0.3 respectively), which also contributes to lower overall biomass. Lourenço et al. (2018) [46] also found similar patterns in biomass with Bijante having significantly higher values than Bruce and Escadinhas. However, the mean biomass values reported in that study for Bijante and Bruce in January were considerably higher than our findings for the same month (8.5 vs 3.71 g AFDM.m^-2^ in Bijante; 5.5 vs 2.08 g AFDM m^-2^ in Bruce), while for Escadinhas, values were similar. These differences may derive from the different methods used to estimate biomass (direct measure vs. estimates from equations in our study) and/or a lower contribution from fiddler crabs in our study because of the sampling method used.

The spatial variation at the (sub)class level in terms of density and biomass was translated in marked differences in the most important (sub)classes between the sites. Furthermore, spatial differences were also apparent in the results of the multivariate analysis, with overall significant differences between sites in the community composition and in community diversity (multivariate dispersal). The aggregated results of all analysis clearly indicate that spatial variation needs to be taken into account to understand the intertidal ecosystem of the Bijagós Archipelago. Spatially heterogeneous intertidal landscapes have been reported in tropical systems elsewhere [83, 87, 88], and in other systems like in the Yellow Sea [89], and in the Wadden Sea [21]. But in temperate and northern intertidal areas, these spatial differences in the macrozoobenthic community structure are typically driven by a few abundant species (e.g. [21, 75, 89, 90]) unlike what was found in the Bijagós Archipelago.

Various macrozoobenthos taxa were found to have high importance in characterizing the community composition of each site. In Adonga, the bivalves *P*. *isocardia*, *Keletistes sp*., *A*. *nymphalis* and *T*. *adansonii* were the main taxa driving the marked differences to the community compositions of the other sites. *Pelecyora isocardia* is a small sized bivalve that constitutes the most important prey item for Red knots *Calidris canutus* in the Bijagós Archipelago, totalling more than 80% of the proportion of biomass consumed by this shorebird [18]. *Pelecyora isocardia* is also important for Bar-tailed godwits *Limosa lapponica*, although to a lesser extent, for which it was found as a prey item in 30% of the droppings analysed in the Bijagós Archipelago [18]. Also, *T*. *adansonii* and *T*. *nymphalis* are consumed to some extent by Red knots and Bar-tailed godwits [18]. Sites like Escadinhas, Bruce and Abu were shown to have macrozoobenthic communities with high influence from different polychaete taxa, which comprise important prey items for several shorebird species [18, 72, 91] and for both juvenile and adult benthic fish [19, 92]. Due to the differences in food preference between different species of these intertidal consumers, the spatial variation in the macrozoobenthic communities found in the Bijagós Archipelago is likely to promote differential space use between the species of these high-level consumers [93–95].

In the CAP analysis we compared the community composition and diversity among sites for each period (i.e. end of wet, early dry and late dry), with significant differences between most sites emerging in all periods. It is noteworthy that Adonga was the site showing the largest separation from the other sites, with the macrozoobenthic community being characterized by high densities of bivalve taxa, and a significantly lower community variability (multivariate dispersal). Moreover, when considering the weighted mean Shannon-Wiener diversity index and species richness in each site, Adonga was also the least diverse and poorest site (although these results should be interpreted cautiously, as sampling there had limited temporal representation; Table 1 in S2 Appendix). On the other hand, Anrumai and Abu were the most similar in the CAP analysis, both in community structure and multivariate dispersal, and also displayed virtually identical species richness and Shannon-Wiener index, having only showed significant differences between each other in the community structure during the early dry season. The similarities between these two sites are also supported by the comparisons between their density and biomass, which never differed significantly for any macrozoobenthos (sub)class. This suggests that these two sites may have similarities in key biotic and abiotic habitat traits and, therefore, in shorebird and benthic fish species assemblages preying on their macrozoobenthic communities. These sites are also the two closest in our sampling, which can be an important factor determining the similarities between their macrozoobenthic communities.

### 4.3. Temporal variation of macrozoobenthos communities

Interestingly, the overall density of macrozoobenthos increased from the end of the wet season to the end of the dry season. After the wet season, the number of individuals started to increase despite the presence of their seasonal predators (shorebirds), suggesting recruitment at this stage [34]. This recruitment hypothesis is further supported by the fact that some species had the highest average body sizes in the late wet season, suggesting the appearance of smaller sized individuals in the population during the course of the dry season. For example, the largest shell sizes of the bivalve *Pelecyora isocardia*, were found at the end of the wet season (7.0 ± 0.2 mm), and the smallest in the dry season (2.5 ± 0.1 mm in the beginning and 3.9 ± 0.1 mm in the end of the season) and the same happened with body sizes of the Orbiniidae polychaetes (20.3 ± 1.3 mm in the end of the wet season vs 17.7 ± 1.1 and 17.5 ± 1.2 in early and late dry season, respectively). These results contrast with the findings from Banc d’Arguin, where the timing of the wet season is similar to that of the Bijagós, but where, next to the Sahara, the magnitude of the wet is of course much smaller. At the Banc d’Arguin intertidal flats most bivalve species densities peaked in October and declined until March, declines which were argued to be explained by depletion by molluscivore shorebirds [29]. In the Bijagós mudflats no significant decreases in density in the course of the shorebird predation season (October-March) were detected, with no evidence for depletion. We propose that the heavy rainfall during the wet season decreases water salinity to an extent that it increases the physiological stress on macrozoobenthos and induces mortality [96]. The subsequent recruitment and high growth rates over the following dry season would, however, ‘overrule’ any depletion effects shown by the (rather more abundant) shorebirds at Banc d’Arguin.

Overall biomass did not vary significantly through time. However, these results must be interpreted with caution, as they were extremely influenced by the dominance of bivalve biomass (72% of total biomass), which remained stable throughout the sampling season. Regardless, no clear pattern in the variation of the majority of (sub)classes emerged, with some sites showing a significant increase in biomass over time, while others showed a decrease. Only gastropod biomass increased consistently across sites, even though values were overall very low. This is likely due to the sampling strategy, as the larger gastropod species have very sparse distribution (e.g. *Pugilina morio*), while some additionally show a preference for mudflat channels (e.g. *Cymbium sp*). Biomass at temperate intertidal sites is typically higher in spring, when macrozoobenthic species’ growth rates are high due to increasing temperatures, and is followed by a decrease until winter [76, 97]. But at tropical sites temperature tends to be more stable throughout the year, and rainfall patterns seem to influence temporal variation the most [96]. Nonetheless, in this study no clear temporal variation of biomass was detected, which may indicate that macrozoobenthos biomass is rather stable throughout the year in the Bijagós Archipelago, as it has been suggested for other tropical sites [98].

We found no clear patterns when assessing the temporal variation in the community composition overall and within each site. While in some sites there was some variation in the most important taxa between periods, the absence of clear patterns between periods is an indication that communities at each site remained rather stable throughout time, and that the spatial variability was higher than the temporal variability, maybe due to site-specific abiotic factors that were not explored in this study, as demonstrated for macroinvertebrate communities elsewhere [76, 99].

The observed increase in density (potentially due to recruitment) and the absence of a decrease in biomass throughout the study period, which coincides with the presence of large numbers of migratory shorebirds wintering in the Bijagós Archipelago, together with the fact that communities remain rather stable between periods, suggests that despite their large numbers, shorebirds do not cause depletion of macroinvertebrates at this site.

## 5. Conclusion

In this work we tackled an evident knowledge gap, by providing a detailed description of the composition, density and biomass of macrozoobenthic communities at a wide spatial-temporal scale within the Bijagós Archipelago. This constitutes an important contribution for the understanding of the intertidal ecosystem of this key biodiversity site, which harbours important benthic fish communities and is a key non-breeding area for Palearctic migratory shorebirds, for which macrozoobenthos constitutes an important prey group. This baseline knowledge will be relevant for subsequent ecological studies in this area focused on the populations of these high-level consumers and on further assessing the interactions within the whole intertidal food web.

Given the high sample size and wide spatial and temporal representativeness, the main findings of this study allow for the conclusion that the macrozoobenthic community of the Bijagós Archipelago is diverse but has relatively low biomass values, is highly variable between sites, and presents densities that increase towards the end of the dry season, despite the high predation pressure by migratory shorebirds during this period. Furthermore, our results suggested that there is a high number of important macrozoobenthic taxa, possibly indicating a resilient intertidal ecosystem. These traits may well be among the important services that the Bijagós Archipelago offers and that may help explain the regional and international importance of this site, for benthic fish in the West African region, and within the East Atlantic Flyway for migratory shorebirds.

## Supporting information

S1 Table Number of samples (sediment cores) collected in each month across sites. (DOCX)

S2 Table Inter-annual comparisons of density and biomass. (DOCX)

S3 Table Mean density (ind.m^2^) and biomass (mg of AFDW.m^2^) and standard error, of the macrozoobenthos taxa found in 609 sediment core samples collected in six intertidal flats of the Bijagós Archipelago. (DOCX)

S4 Table Post-hoc pairwise contrasts of density and biomass between in each site. (DOCX)

S5 Table Post-hoc Tukey HSD tests between sites for multivariate dispersion. (DOCX)

S6 Table Post-hoc PERMANOVAs comparing sites within each period. (DOCX)

S1 Fig Canonical analysis of principal components based on discriminant analysis with all species displayed. (DOCX)

S1 Appendix Macrozoobenthos photographic reference collection. (DOCX)

S2 Appendix Spatial variation in species richness and diversity. (DOCX)

S1 Checklist Questionnaire on inclusivity in global research. (DOCX)
