# Supplementary material for: Spatial and seasonal variation in macrozoobenthic density, biomass and community composition in a major tropical intertidal area, the Bijagós Archipelago, West-Africa
Source: PLoS One. 2022 Nov 28;17(11):e0277861. doi: 10.1371/journal.pone.0277861 (PMC9704600; doi:10.1371/journal.pone.0277861)
Supplement: S1 Table — (DOCX) [file pone.0277861.s001.docx]

**Table S1. Number of samples (sediment cores) collected in each month across sites**

| **Year/month** | **Period** | **Island of Formosa** | | **Island of Bubaque** | | | **Island of Orango** | **Total** |
| --- | --- | --- | --- | --- | --- | --- | --- | --- |
|  |  | **Anrumai** | **Abu** | **Bijante** | **Bruce** | **Escadinhas** | **Adonga** |  |
| **2018** |  | **48** | **47** | **68** | **68** | **68** | **21** | **320** |
| January | Early dry season | 8 | 7 | 0 | 0 | 0 | 0 | 15 |
| February |  | 16 | 16 | 16 | 16 | 16 | 0 | 80 |
| March | Late dry season | 0 | 0 | 16 | 16 | 16 | 0 | 48 |
| October | End of wet season | 12 | 0 | 12 | 12 | 12 | 21 | 69 |
| November |  | 0 | 12 | 12 | 12 | 12 | 0 | 48 |
| December | Early dry season | 12 | 12 | 12 | 12 | 12 | 0 | 60 |
| **2019** |  | **24** | **24** | **24** | **24** | **24** | **87** | **187** |
| January | Early dry season | 0 | 0 | 12 | 12 | 12 | 0 | 36 |
| February |  | 0 | 0 | 0 | 0 | 0 | 20 | 0 |
| March | Late dry season | 12 | 12 | 0 | 0 | 0 | 31 | 55 |
| April |  | 12 | 12 | 12 | 12 | 12 | 0 | 60 |
| October | End of wet season | 0 | 0 | 0 | 0 | 0 | 36 | 36 |
| **2020** |  | **12*** | **24*** | **24*** | **0** | **24*** | **0** | **84*** |
| January | Early dry season | 0 | 0 | 12* | 0 | 12* | 0 | 24* |
| February |  | 12* | 12* | 0 | 0 | 0 | 0 | 24* |
| March | Late dry season | 0 | 12* | 12* | 0 | 12* | 0 | 36* |
| **Total** | | **84** | **95** | **116** | **92** | **116** | **108** | **591** |

Number of samples (sediment cores) collected in each month between 2018 and 2020, across sites (n = 6) in the islands of Formosa, Bubaque and Orango, of the Bijagós Archipelago, Guinea-Bissau. * refers to cores collected in 2020, in which polychaetes were not sorted and were therefore excluded from diversity and community analysis.
