# Supplementary material for: Spatial and seasonal variation in macrozoobenthic density, biomass and community composition in a major tropical intertidal area, the Bijagós Archipelago, West-Africa
Source: PLoS One. 2022 Nov 28;17(11):e0277861. doi: 10.1371/journal.pone.0277861 (PMC9704600; doi:10.1371/journal.pone.0277861)
Supplement: S2 Table — (DOCX) [file pone.0277861.s002.docx]

**Table S2. Inter-annual comparisons of density and biomass.**

| Month | October | | | January | | | February | | | March | | |
| --- | --- | --- | --- | --- | --- | --- | --- | --- | --- | --- | --- | --- |
|  | n | W | p | n | W | p | n | W | p | n | W | p |
| Anrumai |  |  |  |  |  |  |  |  |  |  |  |  |
| Bivalvia | - | - | - | - | - | - | 28 | 72 | 0.3 | - | - | - |
| Gastropoda | - | - | - | - | - | - | 28 | 107.5 | 0.5 | - | - | - |
| Malacostraca | - | - | - | - | - | - | 28 | 91 | 0.8 | - | - | - |
| Abu |  |  |  |  |  |  |  |  |  |  |  |  |
| Bivalvia | - | - | - | - | - | - | 28 | 61 | 0.1 | 24 | 60 | 0.5 |
| Gastropoda | - | - | - | - | - | - | 28 | 94 | 0.9 | 24 | 66 | 0.7 |
| Malacostraca | - | - | - | - | - | - | 28 | 128.5 | 0.1 | 24 | 54 | 0.3 |
| Bijante |  |  |  |  |  |  |  |  |  |  |  |  |
| Bivalvia | - | - | - | 24 | 96.5 | 0.2 | - | - | - | 28 | 122 | 0.2 |
| Gastropoda | - | - | - | 24 | 79.5 | 0.7 | - | - | - | 28 | 91 | 0.8 |
| Malacostraca | - | - | - | 24 | 90.5 | 0.3 | - | - | - | 28 | 49.5 | **0.03** |
| Escadinhas |  |  |  |  |  |  |  |  |  |  |  |  |
| Bivalvia | - | - | - | 24 | 63.5 | 0.6 | - | - | - | 28 | 78 | 0.4 |
| Gastropoda | - | - | - | 24 | 66 | 0.4 | - | - | - | 28 | 48 | **0.008** |
| Malacostraca | - | - | - | 24 | 101.5 | **0.04** | - | - | - | 28 | 89 | 0.7 |
| Adonga |  |  |  |  |  |  |  |  |  |  |  |  |
| Bivalvia | 57 | 130 | **<0.001** | - | - | - | - | - | - | - | - | - |
| Gastropoda | 57 | 346.5 | 0.2 | - | - | - | - | - | - | - | - | - |
| Malacostraca | 57 | 379 | 1 | - | - | - | - | - | - | - | - | - |
| Polychaeta sedentaria | 57 | 227 | **0.01** | - | - | - | - | - | - | - | - | - |
| Polychaeta errantia | 57 | 279 | 0.1 | - | - | - | - | - | - | - | - | - |

Inter-seasonal comparison of densities (ind.m-2) measured in cores collected in the same month of two different years, within each site and for each macrozoobenthos (sub)class. This aims at validating the methodological decision of pulling together sediment cores from different years (2018, 2019 and 2020) to build a continuous data set throughout the months. In all cases, months were sampled a second time in 2020, when polychaetes were not collected in most sites (due to the Covid-19 pandemic), therefore, comparisons were made for the remaining macrozoobenthos (sub)classes; exception was in Adonga, where all (sub)classes were collected. November and April were never sampled twice and were therefore excluded from this analysis. Normality assumptions were tested using Shapiro-Wilk normality test, and comparisons were made using two-sample Wilcoxon tests with a 95% confidence interval. Significant results are marked in bolt.
