# Supplementary material for: Spatial and seasonal variation in macrozoobenthic density, biomass and community composition in a major tropical intertidal area, the Bijagós Archipelago, West-Africa
Source: PLoS One. 2022 Nov 28;17(11):e0277861. doi: 10.1371/journal.pone.0277861 (PMC9704600; doi:10.1371/journal.pone.0277861)
Supplement: S3 Table — (DOCX) [file pone.0277861.s003.docx]

**Table S3. Mean density (ind.m^2^) and biomass (mg of AFDW.m^2^) and standard error, of the macrozoobenthos taxa found in 609 sediment core samples collected in six intertidal flats of the Bijagós Archipelago, in Guinea-Bissau, West Africa.** Overall, at least 88 taxa were identified in this study, at different taxonomic levels. These were aggregated into 82 taxa for the diversity, biomass, and community analysis, to avoid taxa repetition. Of the 88 identified taxa, 24 were identified to the species level, 32 to genus, 21 to family, 5 to order, 1 to suborder, 1 to infraorder and 4 remained as unidentified morphotypes (marked with #). Macrozoobenthos were identified to the lowest taxonomical level possible using a field reference guide built during this work. Taxonomic names followed the World Register of Marine Species. * represents taxa for which we were able to identify some individuals to lower taxonomical levels or to distinguish a particular morphotype, but were pooled into superior taxonomic levels to measure density and biomass.

| (Sub) Class | Family | Taxa | OVERALL | | | | Anrumei | | | | Abu | | | | Bijante | | | | Bruce | | | | Escadinhas | | | | Adonga | | | |
| --- | --- | --- | --- | --- | --- | --- | --- | --- | --- | --- | --- | --- | --- | --- | --- | --- | --- | --- | --- | --- | --- | --- | --- | --- | --- | --- | --- | --- | --- | --- |
|  |  |  | Dens | | Biom | | Dens | | Biom | | Dens | | Biom | | Dens | | Biom | | Dens | | Biom | | Dens | | Biom | | Dens | | Biom | |
|  |  |  | mean | SE | mean | SE | mean | SE | mean | SE | mean | SE | mean | SE | mean | SE | mean | SE | mean | SE | mean | SE | mean | SE | mean | SE | mean | SE | mean | SE |
| Bivalvia | Arcidae | Senilia senilis | 56.2 | 5.7 | 981.7 | 84.0 | 62.2 | 17.1 | 1439.7 | 289.3 | 96.5 | 17.8 | 2377.9 | 322.3 | 127.4 | 19.8 | 732.5 | 139.0 | 11.1 | 5.1 | 191.2 | 81.8 | 7.7 | 2.8 | 40.1 | 26.2 | 30.3 | 5.8 | 972.9 | 165.2 |
|  | Corbulidae | Corbula cadenati | 0.2 | 0.2 | NA | NA | 1.5 | 1.5 | NA | NA | 0 | 0 | NA | NA | 0 | 0 | NA | NA | 0 | 0 | NA | NA | 0 | 0 | NA | NA | 0 | 0 | NA | NA |
|  | Corbulidae | Corbula chudeaui | 3.8 | 1.0 | NA | NA | 9.1 | 4.2 | NA | NA | 9.4 | 4.4 | NA | NA | 2.2 | 1.5 | NA | NA | 2.8 | 1.9 | NA | NA | 1.1 | 1.1 | NA | NA | 0 | 0 | NA | NA |
|  | Corbulidae | Corbula laticostata | 1.9 | 0.7 | NA | NA | 1.5 | 1.5 | NA | NA | 6.7 | 3.5 | NA | NA | 1.1 | 1.1 | NA | NA | 1.4 | 1.4 | NA | NA | 1.1 | 1.1 | NA | NA | 0 | 0 | NA | NA |
|  | Cuspidariidae | Cuspidaria | 0.2 | 0.2 | NA | NA | 0 | 0 | NA | NA | 1.3 | 1.3 | NA | NA | 0 | 0 | NA | NA | 0 | 0 | NA | NA | 0 | 0 | NA | NA | 0 | 0 | NA | NA |
|  | Lasaeidae | Mysella sp | 2.9 | 1.6 | NA | NA | 12.1 | 10.7 | NA | NA | 1.3 | 1.3 | NA | NA | 1.1 | 1.1 | NA | NA | 5.5 | 3.4 | NA | NA | 0 | 0 | NA | NA | 0 | 0 | NA | NA |
|  | Lasaeidae | Pseudopythina nicklesi | 0.1 | 0.1 | NA | NA | 0 | 0 | NA | NA | 0 | 0 | NA | NA | 0 | 0 | NA | NA | 0 | 0 | NA | NA | 0 | 0 | NA | NA | 0.8 | 0.8 | NA | NA |
|  | Lucinidae | Divaricella chavani | 0.2 | 0.2 | NA | NA | 0 | 0 | NA | NA | 1.3 | 1.3 | NA | NA | 0 | 0 | NA | NA | 0 | 0 | NA | NA | 0 | 0 | NA | NA | 0 | 0 | NA | NA |
|  | Lucinidae | Keletistes sp | 43.2 | 4.2 | 45.8 | 4.8 | 62.2 | 12.7 | 73.9 | 13.5 | 77.8 | 16.3 | 97.1 | 18.2 | 15.4 | 5.4 | 13.8 | 6.3 | 1.4 | 1.4 | 0.5 | 0.5 | 7.7 | 3.2 | 7.7 | 3.7 | 101.6 | 12.3 | 64.4 | 10.8 |
|  | Lucinidae | Lamylucina gaini | 0.4 | 0.3 | NA | NA | 0 | 0 | NA | NA | 0 | 0 | NA | NA | 1.1 | 1.1 | NA | NA | 1.4 | 1.4 | NA | NA | 0 | 0 | NA | NA | 0 | 0 | NA | NA |
|  | Myidae | Cryptomya africana | 6.6 | 1.8 | NA | NA | 0 | 0 | NA | NA | 6.7 | 2.9 | NA | NA | 4.4 | 2.2 | NA | NA | 24.9 | 10.5 | NA | NA | 4.4 | 2.7 | NA | NA | 0.8 | 0.8 | NA | NA |
|  | Mytilidae | Arcuatula elegans | 5.4 | 1.0 | NA | NA | 1.5 | 1.5 | NA | NA | 5.4 | 2.6 | NA | NA | 2.2 | 1.5 | NA | NA | 5.5 | 2.7 | NA | NA | 3.3 | 1.9 | NA | NA | 13.9 | 3.5 | NA | NA |
|  | NID | Carditidae | 0.1 | 0.1 | NA | NA | 0 | 0 | NA | NA | 0 | 0 | NA | NA | 0 | 0 | NA | NA | 0 | 0 | NA | NA | 0 | 0 | NA | NA | 0.8 | 0.8 | NA | NA |
|  | NID | Bivalvia NID | 0.2 | 0.2 | NA | NA | 0 | 0 | NA | NA | 0 | 0 | NA | NA | 0 | 0 | NA | NA | 1.4 | 1.4 | NA | NA | 0 | 0 | NA | NA | 0 | 0 | NA | NA |
|  | NID | Bivalvia B1 (#) | 0.1 | 0.1 | NA | NA | 0 | 0 | NA | NA | 0 | 0 | NA | NA | 0 | 0 | NA | NA | 0 | 0 | NA | NA | 0 | 0 | NA | NA | 0.8 | 0.8 | NA | NA |
|  | NID | Invasora2 (#) | 0.2 | 0.2 | NA | NA | 0 | 0 | NA | NA | 0 | 0 | NA | NA | 1.1 | 1.1 | NA | NA | 0 | 0 | NA | NA | 0 | 0 | NA | NA | 0 | 0 | NA | NA |
|  | NID | Newspbiv (#) | 0.4 | 0.3 | NA | NA | 0 | 0 | NA | NA | 2.7 | 1.9 | NA | NA | 0 | 0 | NA | NA | 0 | 0 | NA | NA | 0 | 0 | NA | NA | 0 | 0 | NA | NA |
|  | Noetiidae | Arcopsis afra | 0.2 | 0.2 | NA | NA | 0 | 0 | NA | NA | 1.3 | 1.3 | NA | NA | 0 | 0 | NA | NA | 0 | 0 | NA | NA | 0 | 0 | NA | NA | 0 | 0 | NA | NA |
|  | Solecurtidae | Tagelus adansonii | 35.1 | 3.6 | 622.3 | 107.4 | 54.6 | 9.7 | 1913.9 | 667.5 | 14.8 | 5.0 | 246.3 | 139.2 | 8.8 | 3.7 | 59.7 | 47.0 | 9.7 | 6.3 | 179.0 | 160.4 | 25.3 | 5.7 | 227.7 | 202.7 | 98.3 | 14.4 | 985.7 | 152.4 |
|  | Tellinidae | Austromacoma nymphalis | 54.1 | 6.1 | 240.9 | 26.3 | 68.2 | 15.3 | 357.6 | 104.6 | 37.5 | 12.0 | 370 | 105.1 | 93.3 | 23.9 | 224.6 | 47.1 | 2.8 | 1.9 | 72.0 | 68.8 | 17.6 | 6.2 | 73.6 | 37.1 | 98.3 | 12.6 | 305.0 | 37.9 |
|  | Tellinidae | Moerella distorta | 15.8 | 2.8 | 11.2 | 2.8 | 9.1 | 4.7 | 5.4 | 3.2 | 59.0 | 14.7 | 19.1 | 4.9 | 19.8 | 5.5 | 24.6 | 11.8 | 9.7 | 3.5 | 16.0 | 9.8 | 1.1 | 1.1 | 0.2 | 0.2 | 0 | 0 | 0 | 0 |
|  | Ungulinidae | Diplodonta dautzenbergi | 13.9 | 2.4 | NA | NA | 22.7 | 8.9 | NA | NA | 8.0 | 3.7 | NA | NA | 34.0 | 9.3 | NA | NA | 11.1 | 4.7 | NA | NA | 3.3 | 1.9 | NA | NA | 4.1 | 2.4 | NA | NA |
|  | Ungulinidae | Diplodonta1 sp | 41.6 | 7.2 | 58.9 | 13.6 | 81.9 | 28.5 | 54.1 | 33.8 | 88.5 | 34.7 | 56.7 | 25.3 | 57.1 | 11.0 | 59.8 | 16.9 | 19.4 | 6.2 | 9.3 | 2.9 | 11.0 | 5.1 | 160.5 | 68.0 | 4.1 | 2.4 | 1.8 | 1.2 |
|  | Ungulinidae | Lamysia jousseaumei | 10.3 | 2.0 | NA | NA | 3.0 | 2.1 | NA | NA | 0 | 0 | NA | NA | 20.9 | 5.4 | NA | NA | 4.2 | 2.4 | NA | NA | 8.8 | 5.1 | NA | NA | 20.5 | 7.1 | NA | NA |
|  | Veneridae | Pelecyora isocardia | 176.1 | 14.1 | 160.2 | 13.3 | 262.4 | 43.4 | 87.4 | 26.5 | 189.1 | 45.7 | 28.1 | 4.9 | 86.8 | 19.7 | 30 | 10.7 | 13.8 | 5.7 | 13.1 | 5.5 | 27.5 | 6.6 | 10.2 | 3.4 | 491.6 | 40.8 | 320.8 | 28.1 |
|  | BIVALVIA | | 469.4 | 23.5 | 4044.9 | 409.2 | 652.1 | 71.0 | 6669.3 | 1817.9 | 607.4 | 77.0 | 7521.5 | 1469.7 | 476.6 | 42.8 | 2954.4 | 648.3 | 126.0 | 20.7 | 693.6 | 386.6 | 119.7 | 16.7 | 576.5 | 289.4 | 866.1 | 58.5 | 6696.8 | 781.2 |
| Polychaeta sedentaria | Arenicolidae | Arenicolidae | 0.2 | 0.2 | NA | NA | 0 | 0 | NA | NA | 0 | 0 | NA | NA | 0 | 0 | NA | NA | 0 | 0 | NA | NA | 0 | 0 | NA | NA | 0.8 | 0.8 | NA | NA |
|  | Capitellidae | Capitellidae | 34.7 | 4.0 | 46.0 | 1.6 | 25.2 | 5.6 | 38.6 | 3.4 | 38.6 | 8.0 | 49.4 | 2.8 | 31.5 | 5.2 | 45.1 | 2.8 | 21.1 | 6.4 | 32.3 | 3.0 | 72.3 | 19.7 | 56.7 | 1.6 | 20.5 | 3.6 | 43.0 | 7.2 |
|  |  | *Capitella sp |  |  |  |  |  |  |  |  |  |  |  |  |  |  |  |  |  |  |  |  |  |  |  |  |  |  |  |  |
|  |  | *Heteromastus filiformis |  |  |  |  |  |  |  |  |  |  |  |  |  |  |  |  |  |  |  |  |  |  |  |  |  |  |  |  |
|  | Chaetopteridae | Spiochaetopterus | 1.2 | 0.6 | NA | NA | 1.8 | 1.8 | NA | NA | 7.2 | 4.3 | NA | NA | 0 | 0 | NA | NA | 0 | 0 | NA | NA | 0 | 0 | NA | NA | 0 | 0 | NA | NA |
|  | Cirratulidae | Cirratulidae | 105.8 | 10.5 | 70.9 | 3.3 | 25.9 | 6.7 | 52.8 | 6.9 | 17.9 | 5.1 | 46.7 | 8.0 | 145.9 | 28.6 | 78.8 | 2.0 | 57.2 | 12.1 | 68.3 | 4.3 | 58.2 | 11.9 | 59.9 | 3.9 | 264.7 | 41.1 | 76.7 | 7.8 |
|  |  | *Cirriformia sp |  |  |  |  |  |  |  |  |  |  |  |  |  |  |  |  |  |  |  |  |  |  |  |  |  |  |  |  |
|  |  | *Kirkegaardia sp |  |  |  |  |  |  |  |  |  |  |  |  |  |  |  |  |  |  |  |  |  |  |  |  |  |  |  |  |
|  | Magelonidae | Magelona cincta | 6.0 | 1.3 | NA | NA | 5.3 | 3.0 | NA | NA | 19.7 | 7.5 | NA | NA | 8.3 | 3.3 | NA | NA | 2.8 | 1.9 | NA | NA | 0 | 0 | NA | NA | 3.3 | 1.6 | NA | NA |
|  | Maldanidae | Maldanidae | 41.3 | 3.8 | 386.7 | 29.6 | 30.1 | 7.0 | 579.4 | 108.8 | 23.3 | 5.0 | 511.8 | 99.0 | 26.8 | 5.2 | 471.7 | 96.6 | 36.5 | 7.7 | 303.0 | 41.9 | 16.2 | 3.9 | 353.3 | 94.5 | 98.3 | 14.9 | 285.7 | 39.3 |
|  |  | *maldanidaea |  |  |  |  |  |  |  |  |  |  |  |  |  |  |  |  |  |  |  |  |  |  |  |  |  |  |  |  |
|  |  | *Petaloproctus sp |  |  |  |  |  |  |  |  |  |  |  |  |  |  |  |  |  |  |  |  |  |  |  |  |  |  |  |  |
|  | NID | Polychaeta sedentaria NID | 75.5 | 7.3 | NA | NA | 92.0 | 19.9 | NA | NA | 70 | 15.5 | NA | NA | 119.1 | 25.9 | NA | NA | 66.5 | 18.2 | NA | NA | 96.9 | 15.9 | NA | NA | 20.5 | 5.7 | NA | NA |
|  | Ophelidae | Armandia sp | 5.3 | 1.5 | NA | NA | 1.8 | 1.8 | NA | NA | 1.8 | 1.8 | NA | NA | 16.6 | 6.9 | NA | NA | 8.3 | 3.8 | NA | NA | 2.8 | 1.9 | NA | NA | 0 | 0 | NA | NA |
|  | Orbiniidae | Orbiniidae | 43.5 | 3.5 | 101.4 | 6.0 | 20.1 | 4.6 | 51.6 | 12.6 | 23.9 | 4.6 | 96.3 | 19.5 | 68.8 | 10.1 | 79.3 | 7.6 | 112.6 | 14.5 | 181.8 | 14.5 | 26.3 | 5.4 | 99.9 | 16.7 | 6.3 | 1.9 | 12.4 | 3.7 |
|  |  | *Leodamas sp |  |  |  |  |  |  |  |  |  |  |  |  |  |  |  |  |  |  |  |  |  |  |  |  |  |  |  |  |
|  |  | *Orbinidae A |  |  |  |  |  |  |  |  |  |  |  |  |  |  |  |  |  |  |  |  |  |  |  |  |  |  |  |  |
|  | Paraonidae | Paraonidae | 20.8 | 1.9 | 43.7 | 4.7 | 13.4 | 3.5 | 45.5 | 16.8 | 14.7 | 3.6 | 59.5 | 13.2 | 44.6 | 7.6 | 59.6 | 14.3 | 26.9 | 5.2 | 62.4 | 7.9 | 5.5 | 1.5 | 16.0 | 4.9 | 17.4 | 3.2 | 13.9 | 3.2 |
|  |  | *Aricidea sp A |  |  |  |  |  |  |  |  |  |  |  |  |  |  |  |  |  |  |  |  |  |  |  |  |  |  |  |  |
|  |  | *Aricidea sp B |  |  |  |  |  |  |  |  |  |  |  |  |  |  |  |  |  |  |  |  |  |  |  |  |  |  |  |  |
|  |  | *Paraonidae A |  |  |  |  |  |  |  |  |  |  |  |  |  |  |  |  |  |  |  |  |  |  |  |  |  |  |  |  |
|  | Spionidae | Prionospio sp | 1.3 | 0.5 | NA | NA | 0 | 0 | NA | NA | 0 | 0 | NA | NA | 1.4 | 1.4 | NA | NA | 4.2 | 2.4 | NA | NA | 0 | 0 | NA | NA | 1.6 | 1.2 | NA | NA |
|  | Terebellidae | Terebellidae | 2.8 | 0.5 | NA | NA | 4.1 | 1.5 | 0 | 0 | 1.2 | 0.8 | NA | NA | 4.6 | 1.7 | NA | NA | 1.8 | 1.1 | NA | NA | 3.2 | 1.4 | NA | NA | 1.9 | 0.8 | NA | NA |
|  |  | *Polycirrus sp |  |  |  |  |  |  |  |  |  |  |  |  |  |  |  |  |  |  |  |  |  |  |  |  |  |  |  |  |
|  |  | *Streblosoma sp |  |  |  |  |  |  |  |  |  |  |  |  |  |  |  |  |  |  |  |  |  |  |  |  |  |  |  |  |
|  | POLYCHAETA SEDENTARIA | | 912.4 | 39.7 | 1088.4 | 68.1 | 509.6 | 50.1 | 1036.8 | 226.2 | 525.7 | 50.5 | 945.3 | 164.2 | 1232.3 | 93.5 | 1252.4 | 186.6 | 925.0 | 80.5 | 1244.3 | 133.4 | 728.3 | 90.4 | 819.8 | 152.9 | 1308.6 | 119.4 | 1173.5 | 144.5 |
| Polychaeta errantia | Amphinomidae | Amphinomidae | 1.3 | 0.5 | NA | NA | 3.5 | 2.5 | NA | NA | 0 | 0 | NA | NA | 1.4 | 1.4 | NA | NA | 0 | 0 | NA | NA | 1.4 | 1.4 | NA | NA | 1.6 | 1.2 | NA | NA |
|  | Eunicidae | Eunicidae | 3.5 | 0.7 | NA | NA | 0 | 0 | NA | NA | 0 | 0 | NA | NA | 0.7 | 0.7 | NA | NA | 1.4 | 1.0 | NA | NA | 0.7 | 0.7 | NA | NA | 14.7 | 2.8 | NA | NA |
|  |  | *Eunice sp |  |  |  |  |  |  |  |  |  |  |  |  |  |  |  |  |  |  |  |  |  |  |  |  |  |  |  |  |
|  | Eunicidae | Marphysa sanguinea | 5.0 | 0.8 | 61.7 | 16.1 | 3.5 | 1.8 | 74.2 | 41.7 | 5.4 | 2.2 | 9.9 | 4.8 | 2.1 | 1.2 | 28.9 | 26.0 | 0 | 0 | 0 | 0 | 7.6 | 2.4 | 69.0 | 42.2 | 10.2 | 2.3 | 140.4 | 51.5 |
|  | Glyceridae | Glycera sp | 53.3 | 3.8 | 74.3 | 11.3 | 84.9 | 14.7 | 73.5 | 12.5 | 53.8 | 11.3 | 135.6 | 77.4 | 72.0 | 8.7 | 97.0 | 14.2 | 48.5 | 8.1 | 72.8 | 15.3 | 40.2 | 8.1 | 44.7 | 10.5 | 31.1 | 5.2 | 44.0 | 8.7 |
|  | Goniadidae | Goniadopsis sp | 14.3 | 2.6 | 7.1 | 0.9 | 7.1 | 4.3 | 3.6 | 1.8 | 10.8 | 4.9 | 5.4 | 2.1 | 33.2 | 10 | 15.7 | 2.8 | 18.0 | 7.3 | 8.7 | 2.3 | 16.6 | 6.0 | 8.2 | 2.2 | 0 | 0 | 0 | 0 |
|  | Hesionidae | Leocrates sp | 3.6 | 1.4 | NA | NA | 0 | 0 | NA | NA | 0 | 0 | NA | NA | 12.5 | 6.5 | NA | NA | 5.5 | 3.4 | NA | NA | 2.8 | 2.8 | NA | NA | 0 | 0 | NA | NA |
|  | Lumbrineridae | Lumbrineridae | 30.1 | 3.8 | 51.6 | 7.5 | 14.2 | 5.4 | 34.2 | 13.8 | 7.2 | 3.5 | 24.5 | 18.5 | 69.2 | 12.4 | 95.6 | 20.1 | 9.7 | 5.3 | 18.1 | 7.5 | 52.6 | 14.8 | 62.4 | 18.9 | 20.5 | 4.1 | 54.6 | 19.8 |
|  | Nephtyidae | Nephtyidae | 7.0 | 1.4 | NA | NA | 5.3 | 3.0 | NA | NA | 0 | 0 | NA | NA | 0 | 0 | NA | NA | 2.8 | 1.9 | NA | NA | 4.2 | 2.4 | NA | NA | 24.6 | 5.4 | NA | NA |
|  | Nereididae | Nereididae | 26.8 | 2.8 | 22.7 | 1.8 | 13.3 | 3.5 | 14.2 | 4.2 | 40.4 | 9.8 | 57.5 | 8.4 | 21.5 | 8.5 | 22.6 | 4.6 | 13.8 | 4.2 | 9.3 | 2.4 | 39.5 | 7.6 | 25.5 | 3.4 | 31.5 | 5.3 | 11.5 | 1.5 |
|  | NID | Phyllodocida1 | 0.2 | 0.2 | NA | NA | 0 | 0 | NA | NA | 0 | 0 | NA | NA | 0 | 0 | NA | NA | 0 | 0 | NA | NA | 0 | 0 | NA | NA | 0.8 | 0.8 | NA | NA |
|  | NID | Polychaeta errantia NID | 3.4 | 0.9 | NA | NA | 7.1 | 4.3 | NA | NA | 0 | 0 | NA | NA | 1.4 | 1.4 | NA | NA | 2.8 | 1.9 | NA | NA | 1.4 | 1.4 | NA | NA | 7.4 | 2.6 | NA | NA |
|  | Oenonidae | Oenonidae | 0.5 | 0.4 | NA | NA | 0 | 0 | NA | NA | 0 | 0 | NA | NA | 0 | 0 | NA | NA | 0 | 0 | NA | NA | 0 | 0 | NA | NA | 2.5 | 1.8 | NA | NA |
|  | Onuphidae | Diopatra sp | 43.4 | 5.3 | 26.1 | 4.0 | 21.2 | 6.2 | 23.0 | 8.1 | 163.3 | 29.4 | 57.0 | 10.7 | 77.5 | 13.6 | 27.4 | 4.1 | 12.5 | 4.0 | 5.2 | 1.9 | 1.4 | 1.4 | 0.1 | 0.1 | 12.3 | 3.4 | 28.9 | 14.6 |
|  | Pilargidae | Pilargidae1 | 0.3 | 0.2 | NA | NA | 0 | 0 | NA | NA | 0 | 0 | NA | NA | 0 | 0 | NA | NA | 0 | 0 | NA | NA | 0 | 0 | NA | NA | 1.6 | 1.2 | NA | NA |
|  | Pilargidae | Sigambra sp | 60.6 | 6.3 | 14.3 | 0.4 | 76.1 | 14.9 | 16.4 | 1.0 | 155.2 | 32.7 | 20.7 | 0.6 | 68.5 | 13.6 | 16.3 | 0.9 | 18.7 | 5.3 | 6.4 | 1.1 | 79.6 | 15.8 | 17.0 | 0.9 | 0.8 | 0.8 | 0.5 | 0.3 |
|  | Sigalionidae | Sigalion sp | 0.2 | 0.2 | NA | NA | 0 | 0 | NA | NA | 1.8 | 1.8 | NA | NA | 0 | 0 | NA | NA | 0 | 0 | NA | NA | 0 | 0 | NA | NA | 0 | 0 | NA | NA |
|  | Syllidae | Syllinae | 24.4 | 3.1 | NA | NA | 24.8 | 10.9 | NA | NA | 14.4 | 6.0 | NA | NA | 37.4 | 9.6 | NA | NA | 9.7 | 5.3 | NA | NA | 5.5 | 3.4 | NA | NA | 48.3 | 7.8 | NA | NA |
|  | POLYCHAETA ERRANTIA | | 373.7 | 17.6 | 296.1 | 25.1 | 353.9 | 33.9 | 272.0 | 48.2 | 653.1 | 74.8 | 431.3 | 103.2 | 490.2 | 45.9 | 351.3 | 43.4 | 177.2 | 23.1 | 127.6 | 19.7 | 380.8 | 42.2 | 261.8 | 49.0 | 265.5 | 18.6 | 348.8 | 75.3 |
| Malacostraca | Alpheidae | Alpheidae | 7.5 | 1.3 | NA | NA | 3.0 | 2.1 | NA | NA | 9.4 | 3.4 | NA | NA | 17.6 | 4.7 | NA | NA | 8.3 | 3.8 | NA | NA | 3.3 | 1.9 | NA | NA | 2.5 | 1.4 | NA | NA |
|  | Ampeliscidae | Ampelisca sp | 12.3 | 4.0 | NA | NA | 0 | 0 | NA | NA | 2.7 | 1.9 | NA | NA | 51.6 | 19.2 | NA | NA | 13.8 | 8.9 | NA | NA | 0 | 0 | NA | NA | 0 | 0 | NA | NA |
|  | Anthuridae | Anthuridae | 14.4 | 2.4 | 16.5 | 1.8 | 13.6 | 4.3 | 16.1 | 5.1 | 24.1 | 7.0 | 26.8 | 5.8 | 7.7 | 3.2 | 9.0 | 3.3 | 23.5 | 10.6 | 25.3 | 5.6 | 13.2 | 5.3 | 14.9 | 4.1 | 7.4 | 2.4 | 8.7 | 2.8 |
|  | Aoridae | Grandidierella sp | 10.6 | 2.7 | 1.2 | 0.2 | 0 | 0 | NA | NA | 0 | 0 | NA | NA | 18.7 | 8.0 | 2.1 | 0.5 | 29.1 | 11.7 | 3.1 | 0.6 | 13.2 | 7.4 | 1.5 | 0.4 | 0.8 | 0.8 | 0.1 | 0.1 |
|  | Callichiridae | Balsscallichirus balssi | 26.1 | 3.5 | 211.1 | 55.5 | 1.5 | 1.5 | 1.5 | 1.5 | 16.1 | 5.5 | 323.9 | 192.8 | 30.7 | 6.9 | 261.5 | 83.8 | 72.0 | 15.4 | 287.4 | 145.5 | 25.3 | 9.5 | 237.9 | 192.9 | 10.7 | 3.4 | 117.3 | 63.2 |
|  | Cirolanidae | Cirolanidae | 0.1 | 0.1 | NA | NA | 0 | 0 | NA | NA | 0 | 0 | NA | NA | 0 | 0 | NA | NA | 0 | 0 | NA | NA | 0 | 0 | NA | NA | 0.8 | 0.8 | NA | NA |
|  | Menippidae | Menippe nodifrons | 0.2 | 0.2 | NA | NA | 0 | 0 | NA | NA | 1.3 | 1.3 | NA | NA | 0 | 0 | NA | NA | 0 | 0 | NA | NA | 0 | 0 | NA | NA | 0 | 0 | NA | NA |
|  | NID | Amphipoda1 | 28.3 | 4.5 | 8.0 | 0.4 | 1.5 | 1.5 | 0.5 | 0.4 | 24.1 | 6.4 | 6.7 | 1.1 | 58.2 | 11.5 | 16.0 | 0.9 | 74.8 | 23.6 | 13.9 | 0.7 | 9.9 | 3.5 | 2.0 | 0.5 | 0.8 | 0.8 | 0.2 | 0.1 |
|  | NID | Amphipoda2 | 7.5 | 2.2 | NA | NA | 0 | 0 | NA | NA | 1.3 | 1.3 | NA | NA | 0 | 0 | NA | NA | 48.5 | 13.6 | NA | NA | 0 | 0 | NA | NA | 0 | 0 | NA | NA |
|  | NID | Amphipoda4 | 0.2 | 0.2 | NA | NA | 0 | 0 | NA | NA | 0 | 0 | NA | NA | 0 | 0 | NA | NA | 1.4 | 1.4 | NA | NA | 0 | 0 | NA | NA | 0 | 0 | NA | NA |
|  | NID | Amphipodatigre | 0.4 | 0.3 | NA | NA | 0 | 0 | NA | NA | 0 | 0 | NA | NA | 0 | 0 | NA | NA | 1.4 | 1.4 | NA | NA | 1.1 | 1.1 | NA | NA | 0 | 0 | NA | NA |
|  | NID | Apseudomorpha | 7.7 | 2.6 | NA | NA | 28.8 | 15.3 | NA | NA | 2.7 | 1.9 | NA | NA | 7.7 | 3.6 | NA | NA | 4.2 | 2.4 | NA | NA | 6.6 | 6.6 | NA | NA | 0 | 0 | NA | NA |
|  | NID | CarideaA (#) | 0.2 | 0.2 | NA | NA | 0 | 0 | NA | NA | 0 | 0 | NA | NA | 0 | 0 | NA | NA | 1.4 | 1.4 | NA | NA | 0 | 0 | NA | NA | 0 | 0 | NA | NA |
|  | NID | Cumacea | 0.4 | 0.3 | NA | NA | 0 | 0 | NA | NA | 0 | 0 | NA | NA | 0 | 0 | NA | NA | 2.8 | 1.9 | NA | NA | 0 | 0 | NA | NA | 0 | 0 | NA | NA |
|  | NID | Decapoda | 0.1 | 0.1 | NA | NA | 0 | 0 | NA | NA | 0 | 0 | NA | NA | 0 | 0 | NA | NA | 0 | 0 | NA | NA | 0 | 0 | NA | NA | 0.8 | 0.8 | NA | NA |
|  | NID | Malacostraca NID | 0.3 | 0.2 | NA | NA | 0 | 0 | NA | NA | 0 | 0 | NA | NA | 0 | 0 | NA | NA | 0 | 0 | NA | NA | 0 | 0 | NA | NA | 1.6 | 1.2 | NA | NA |
|  | Ocypodidae | Afruca tangeri | 17.2 | 2.7 | 113.6 | 33.9 | 7.6 | 3.9 | 21.0 | 19.0 | 10.7 | 4.1 | 25.2 | 13.1 | 12.1 | 4.7 | 83.2 | 71.6 | 4.2 | 2.4 | 25.6 | 23.7 | 52.7 | 11.7 | 404.3 | 140.8 | 9.0 | 3.9 | 4.4 | 1.8 |
|  | Pinnotheridae | Pinnotheridae | 7.3 | 1.5 | NA | NA | 1.5 | 1.5 | NA | NA | 5.4 | 3.3 | NA | NA | 17.6 | 4.7 | NA | NA | 19.4 | 6.8 | NA | NA | 0 | 0 | NA | NA | 0 | 0 | NA | NA |
|  | Portunidae | Callinectes marginatus | 1.8 | 0.6 | NA | NA | 6.1 | 3.0 | NA | NA | 0 | 0 | NA | NA | 2.2 | 1.5 | NA | NA | 1.4 | 1.4 | NA | NA | 1.1 | 1.1 | NA | NA | 0.8 | 0.8 | NA | NA |
|  | MALACOSTRACA | | 146.1 | 10.8 | 391.3 | 75.0 | 68.2 | 16.5 | 39.8 | 20.2 | 101.9 | 13.3 | 402.2 | 199.0 | 230.6 | 27.8 | 422.2 | 120.7 | 308.8 | 47.0 | 472.1 | 243.4 | 128.5 | 19.8 | 764.0 | 267.4 | 35.2 | 6.5 | 152.8 | 74.7 |
| Gastropoda | Bullidae | Bulla sp | 2.1 | 0.7 | NA | NA | 1.5 | 1.5 | NA | NA | 5.4 | 3.3 | NA | NA | 3.3 | 1.9 | NA | NA | 2.8 | 1.9 | NA | NA | 0 | 0 | NA | NA | 0 | 0 | NA | NA |
|  | Cylichnidae | Cylichnidae | 8.4 | 1.8 | NA | NA | 9.1 | 3.6 | NA | NA | 4.0 | 2.3 | NA | NA | 30.7 | 8.3 | NA | NA | 2.8 | 1.9 | NA | NA | 0 | 0 | NA | NA | 1.6 | 1.2 | NA | NA |
|  | Haminoeidae | Haminoea sp | 8.6 | 1.6 | NA | NA | 12.1 | 5.1 | NA | NA | 16.1 | 5.8 | NA | NA | 8.8 | 3.4 | NA | NA | 1.4 | 1.4 | NA | NA | 3.3 | 1.9 | NA | NA | 10.7 | 4.2 | NA | NA |
|  | Hydrobiidae | Hydrobiidae | 8.9 | 3.0 | NA | NA | 4.5 | 3.4 | NA | NA | 6.7 | 6.7 | NA | NA | 22.0 | 13.0 | NA | NA | 8.3 | 5.8 | NA | NA | 3.3 | 3.3 | NA | NA | 6.6 | 3.8 | NA | NA |
|  | Iravadiidae | Hyala sp | 41.5 | 5.1 | 3.8 | 0.2 | 84.9 | 17.0 | 7.2 | 0.5 | 77.8 | 18.4 | 6.5 | 0.5 | 59.3 | 15.2 | 5.3 | 0.4 | 4.2 | 2.2 | 0.5 | 0.2 | 25.3 | 9.6 | 2.7 | 0.4 | 5.7 | 2.1 | 0.6 | 0.2 |
|  | Marginellidae | Prunum sp | 7.3 | 1.7 | NA | NA | 1.5 | 1.5 | NA | NA | 16.1 | 6.4 | NA | NA | 23.1 | 6.7 | NA | NA | 1.4 | 1.4 | NA | NA | 0 | 0 | NA | NA | 0 | 0 | NA | NA |
|  | NID | Pattela (#) | 0.2 | 0.2 | NA | NA | 0 | 0 | NA | NA | 1.3 | 1.3 | NA | NA | 0 | 0 | NA | NA | 0 | 0 | NA | NA | 0 | 0 | NA | NA | 0 | 0 | NA | NA |
|  | Skeneidae | Skeneidae | 0.6 | 0.3 | NA | NA | 1.5 | 1.5 | NA | NA | 0 | 0 | NA | NA | 1.1 | 1.1 | NA | NA | 0 | 0 | NA | NA | 0 | 0 | NA | NA | 0.8 | 0.8 | NA | NA |
|  | Solariellidae | Solariella sp | 23.8 | 4.2 | 8.4 | 0.7 | 3.0 | 2.1 | 1.2 | 0.8 | 33.5 | 12.1 | 11.7 | 2.1 | 30.7 | 7.1 | 11.0 | 1.8 | 69.2 | 19.2 | 19.5 | 2.2 | 9.9 | 8.8 | 3.6 | 1.2 | 0 | 0 | 0 | 0 |
|  | Terebridae | Terebridae | 0.8 | 0.4 | NA | NA | 1.5 | 1.5 | NA | NA | 2.7 | 1.9 | NA | NA | 1.1 | 1.1 | NA | NA | 0 | 0 | NA | NA | 0 | 0 | NA | NA | 0 | 0 | NA | NA |
|  | Tornidae | Cochliolepis sp | 1.3 | 0.6 | NA | NA | 4.5 | 3.4 | NA | NA | 0 | 0 | NA | NA | 2.2 | 1.5 | NA | NA | 1.4 | 1.4 | NA | NA | 0 | 0 | NA | NA | 0 | 0 | NA | NA |
|  | Turritellidae | Turritella sp | 1.3 | 0.5 | NA | NA | 0 | 0 | NA | NA | 1.3 | 1.3 | NA | NA | 5.5 | 2.4 | NA | NA | 0 | 0 | NA | NA | 0 | 0 | NA | NA | 0 | 0 | NA | NA |
|  | GASTROPODA | | 146.0 | 16.5 | 19.2 | 2.4 | 209.3 | 50.9 | 21.6 | 5.8 | 242.7 | 54.6 | 31.6 | 7.8 | 247.1 | 50.0 | 26.1 | 5.7 | 95.5 | 22.4 | 27.8 | 7.4 | 67.0 | 31.7 | 9.9 | 4.7 | 31.1 | 8.6 | 1.9 | 1.0 |
