# Supplementary material for: Spatial and seasonal variation in macrozoobenthic density, biomass and community composition in a major tropical intertidal area, the Bijagós Archipelago, West-Africa
Source: PLoS One. 2022 Nov 28;17(11):e0277861. doi: 10.1371/journal.pone.0277861 (PMC9704600; doi:10.1371/journal.pone.0277861)
Supplement: S4 Table — (DOCX) [file pone.0277861.s004.docx]

**Table S4. Post-hoc pairwise contrasts of density and biomass between in each site following GLM negative binomial models for the overall macrozoobenthic community and for each macrozoobenthos (sub)class.**

|  | **Overall** | | | | **Bivalvia** | | | | **Polychaeta sedentaria** | | | | **Polychaeta errantia** | | | | **Malacostraca** | | | | **Gastropoda** | | | |
| --- | --- | --- | --- | --- | --- | --- | --- | --- | --- | --- | --- | --- | --- | --- | --- | --- | --- | --- | --- | --- | --- | --- | --- | --- |
|  | Estimate | SE | Z | p | Estimate | SE | Z | p | Estimate | SE | Z | p | Estimate | SE | Z | p | Estimate | SE | Z | p | Estimate | SE | Z | p |
| **Contrasts** | **DENSITY** | | | | | | | | | | | | | | | | | | | | | | | |
| Abu - Anrumai | 0.08 | 0.11 | 0.75 | 0.98 | -0.12 | 0.14 | -0.85 | 0.96 | 0.19 | 0.39 | 0.50 | 1.00 | 0.61 | 0.38 | 1.63 | 0.57 | -0.36 | 0.69 | -0.52 | 1.00 | 0.77 | 0.95 | 0.81 | 0.96 |
| Bijante – Anrumai | 0.33 | 0.10 | 3.26 | **0.01** | -0.30 | 0.14 | -2.21 | 0.23 | 0.71 | 0.32 | 2.22 | 0.22 | -0.56 | 0.34 | -1.64 | 0.56 | -0.23 | 0.57 | -0.41 | 1.00 | 0.36 | 0.82 | 0.44 | 1.00 |
| Bruce – Anrumai | -0.02 | 0.11 | -0.182 | 1.00 | -1.64 | 0.17 | -9.54 | **<0.001** | 0.09 | 0.32 | 0.27 | 1.00 | -1.29 | 0.39 | -3.33 | **0.01** | 0.22 | 0.56 | 0.39 | 1.00 | -1.61 | 0.92 | -1.76 | 0.49 |
| Escadinhas – Anrumai | -0.34 | 0.10 | -3.228 | **0.02** | -1.68 | 0.16 | -10.46 | **<0.001** | -0.57 | 0.33 | -1.71 | 0.51 | -1.17 | 0.36 | -3.22 | **0.02** | 0.17 | 0.57 | 0.30 | 1.00 | 0.30 | 0.84 | 0.36 | 1.00 |
| Adonga – Anrumai | 0.48 | 0.10 | 4.578 | **<0.001** | 0.44 | 0.14 | 3.22 | **0.02** | 0.88 | 0.28 | 3.12 | **0.02** | -0.39 | 0.30 | -1.29 | 0.78 | -1.06 | 0.55 | -1.93 | 0.37 | -3.13 | 0.94 | -3.31 | **0.01** |
| Bijante – Abu | 0.25 | 0.10 | 2.563 | 0.11 | -0.18 | 0.13 | -1.37 | 0.74 | 0.52 | 0.35 | 1.46 | 0.68 | -1.18 | 0.35 | -3.32 | **0.01** | 0.13 | 0.62 | 0.21 | 1.00 | -0.41 | 0.87 | -0.48 | 1.00 |
| Bruce – Abu | -0.10 | 0.11 | -0.952 | 0.93 | -1.51 | 0.17 | -8.98 | **<0.001** | -0.10 | 0.36 | -0.29 | 1.00 | -1.90 | 0.40 | -4.80 | <0.001 | 0.58 | 0.61 | 0.95 | 0.93 | -2.38 | 0.96 | -2.47 | 0.13 |
| Escadinhas – Abu | -0.42 | 0.10 | -4.144 | **<0.001** | -1.56 | 0.16 | -9.90 | **<0.001** | -0.76 | 0.37 | -2.08 | 0.29 | -1.78 | 0.37 | -4.78 | <0.001 | 0.53 | 0.62 | 0.86 | 0.95 | -0.47 | 0.89 | -0.53 | 1.00 |
| Adonga – Abu | 0.40 | 0.10 | 3.912 | **0.001** | 0.56 | 0.13 | 4.18 | **<0.001** | 0.69 | 0.32 | 2.15 | 0.26 | -1.00 | 0.31 | -3.22 | **0.02** | -0.70 | 0.60 | -1.16 | 0.85 | -3.90 | 0.99 | -3.94 | **0.001** |
| Bruce – Bijante | -0.35 | 0.10 | -3.54 | **0.01** | -1.33 | 0.16 | -8.08 | **<0.001** | -0.62 | 0.28 | -2.20 | 0.23 | -0.72 | 0.36 | -1.98 | 0.34 | 0.45 | 0.46 | 0.97 | 0.92 | -1.97 | 0.83 | -2.36 | 0.17 |
| Escadinhas – Bijante | -0.67 | 0.09 | -7.059 | **<0.001** | -1.37 | 0.15 | -8.97 | **<0.001** | -1.28 | 0.29 | -4.38 | **<0.001** | -0.60 | 0.34 | -1.78 | 0.47 | 0.40 | 0.48 | 0.85 | 0.96 | -0.06 | 0.75 | -0.08 | 1.00 |
| Adonga – Bijante | 0.15 | 0.09 | 1.541 | 0.64 | 0.74 | 0.13 | 5.84 | **<0.001** | 0.17 | 0.23 | 0.74 | 0.98 | 0.18 | 0.27 | 0.67 | 0.98 | -0.83 | 0.45 | -1.85 | 0.43 | -3.48 | 0.86 | -4.03 | **<0.001** |
| Escadinhas – Bruce | -0.32 | 0.10 | -3.111 | **0.02** | -0.04 | 0.18 | -0.22 | 1.00 | -0.66 | 0.30 | -2.21 | 0.23 | 0.12 | 0.38 | 0.32 | 1.00 | -0.04 | 0.47 | -0.10 | 1.00 | 1.91 | 0.86 | 2.22 | 0.23 |
| Adonga – Bruce | 0.50 | 0.10 | 4.922 | **<0.001** | 2.08 | 0.16 | 12.69 | **<0.001** | 0.79 | 0.24 | 3.30 | **0.01** | 0.90 | 0.32 | 2.80 | 0.06 | -1.27 | 0.44 | -2.92 | **0.04** | -1.52 | 0.96 | -1.58 | 0.61 |
| Adonga – Escadinhas | 0.82 | 0.10 | 8.399 | **<0.001** | 2.12 | 0.15 | 13.89 | **<0.001** | 1.45 | 0.25 | 5.77 | <0.001 | 0.78 | 0.29 | 2.68 | 0.08 | -1.23 | 0.45 | -2.72 | 0.07 | -3.43 | 0.89 | -3.85 | **0.002** |
| **Contrasts** | **BIOMASS** | | | | | | | | | | | | | | | | | | | | | | | |
| Abu - Anrumai | 0.13 | 0.19 | 0.70 | 0.98 | 0.15 | 0.32 | 0.48 | 1.00 | -1.02 | 0.56 | -1.83 | 0.44 | 0.41 | 0.57 | 0.73 | 0.98 | -2.97 | 1.21 | -2.46 | 0.13 | 0.34 | 0.36 | 0.96 | 0.93 |
| Bijante – Anrumai | -0.52 | 0.18 | -2.83 | **0.05** | -0.80 | 0.30 | -2.66 | **0.08** | -0.57 | 0.47 | -1.22 | 0.82 | -0.92 | 0.50 | -1.85 | 0.43 | 0.59 | 0.99 | 0.59 | 0.99 | 0.25 | 0.36 | 0.69 | 0.98 |
| Bruce – Anrumai | -1.12 | 0.19 | -5.76 | **<0.001** | -2.36 | 0.32 | -7.35 | **<0.001** | -0.22 | 0.47 | -0.47 | 1.00 | -1.65 | 0.53 | -3.10 | **0.02** | -1.28 | 1.01 | -1.27 | 0.80 | 0.33 | 0.37 | 0.88 | 0.95 |
| Escadinhas – Anrumai | -1.27 | 0.18 | -6.88 | **<0.001** | -2.50 | 0.30 | -8.22 | **<0.001** | -0.32 | 0.47 | -0.69 | 0.98 | -1.21 | 0.51 | -2.40 | 0.15 | 1.16 | 0.99 | 1.18 | 0.84 | -0.72 | 0.44 | -1.64 | 0.55 |
| Adonga – Anrumai | 0.06 | 0.19 | 0.34 | 1.00 | -0.12 | 0.31 | -0.38 | 1.00 | 0.27 | 0.41 | 0.66 | 0.99 | -0.83 | 0.43 | -1.93 | 0.37 | -2.59 | 0.93 | -2.80 | 0.06 | -2.16 | 0.72 | -2.99 | **0.03** |
| Bijante – Abu | -0.65 | 0.18 | -3.68 | **0.003** | -0.95 | 0.29 | -3.27 | **0.01** | 0.45 | 0.52 | 0.85 | 0.96 | -1.33 | 0.54 | -2.49 | 0.12 | 3.56 | 1.07 | 3.32 | **0.01** | -0.10 | 0.31 | -0.31 | 1.00 |
| Bruce – Abu | -1.25 | 0.19 | -6.64 | **<0.001** | -2.51 | 0.31 | -8.04 | **<0.001** | 0.80 | 0.52 | 1.52 | 0.64 | -2.06 | 0.57 | -3.62 | **0.004** | 1.69 | 1.10 | 1.54 | 0.63 | -0.02 | 0.33 | -0.05 | 1.00 |
| Escadinhas – Abu | -1.40 | 0.18 | -7.86 | **<0.001** | -2.65 | 0.29 | -9.00 | **<0.001** | 0.70 | 0.53 | 1.32 | 0.77 | -1.63 | 0.54 | -2.99 | **0.03** | 4.13 | 1.07 | 3.86 | **0.001** | -1.07 | 0.41 | -2.63 | 0.08 |
| Adonga – Abu | -0.07 | 0.19 | -0.38 | 1.00 | -0.27 | 0.30 | -0.88 | 0.95 | 1.29 | 0.47 | 2.73 | 0.07 | -1.24 | 0.47 | -2.62 | 0.09 | 0.38 | 1.02 | 0.38 | 1.00 | -2.50 | 0.70 | -3.56 | **0.004** |
| Bruce – Bijante | -0.60 | 0.18 | -3.34 | **0.01** | -1.55 | 0.30 | -5.23 | **<0.001** | 0.35 | 0.43 | 0.82 | 0.96 | -0.73 | 0.50 | -1.46 | 0.68 | -1.87 | 0.85 | -2.20 | 0.23 | 0.08 | 0.32 | 0.25 | 1.00 |
| Escadinhas – Bijante | -0.75 | 0.17 | -4.43 | **<0.001** | -1.70 | 0.28 | -6.08 | **<0.001** | 0.25 | 0.43 | 0.58 | 0.99 | -0.30 | 0.47 | -0.63 | 0.99 | 0.57 | 0.82 | 0.70 | 0.98 | -0.97 | 0.40 | -2.40 | 0.15 |
| Adonga – Bijante | 0.58 | 0.17 | 3.35 | **0.01** | 0.69 | 0.29 | 2.40 | 0.16 | 0.84 | 0.36 | 2.31 | 0.18 | 0.09 | 0.39 | 0.23 | 1.00 | -3.18 | 0.74 | -4.28 | **<0.001** | -2.41 | 0.70 | -3.44 | **0.01** |
| Escadinhas – Bruce | -0.15 | 0.18 | -0.83 | 0.96 | -0.14 | 0.30 | 0.48 | 1.00 | -0.10 | 0.43 | 0.24 | 1.00 | 0.44 | 0.51 | -0.86 | 0.96 | 2.44 | 0.85 | -2.89 | **0.04** | -1.05 | 0.42 | 2.52 | 0.11 |
| Adonga – Bruce | 1.18 | 0.18 | 6.42 | **<0.001** | 2.24 | 0.30 | 7.36 | **<0.001** | 0.49 | 0.36 | 1.35 | 0.75 | 0.82 | 0.43 | 1.89 | 0.40 | -1.31 | 0.78 | -1.69 | 0.53 | -2.49 | 0.71 | -3.52 | **0.01** |
| Adonga – Escadinhas | 1.33 | 0.17 | 7.63 | **<0.001** | 2.38 | 0.29 | 8.26 | **<0.001** | 0.59 | 0.36 | 1.62 | 0.58 | 0.38 | 0.40 | 0.96 | 0.93 | -3.75 | 0.74 | -5.08 | **<0.001** | -1.44 | 0.75 | -1.92 | 0.37 |
