# Supplementary material for: Spatial and seasonal variation in macrozoobenthic density, biomass and community composition in a major tropical intertidal area, the Bijagós Archipelago, West-Africa
Source: PLoS One. 2022 Nov 28;17(11):e0277861. doi: 10.1371/journal.pone.0277861 (PMC9704600; doi:10.1371/journal.pone.0277861)
Supplement: S5 Table — (DOCX) [file pone.0277861.s005.docx]

**Table S5:** **Post-hoc Tukey HSD tests with pairwise comparisons between sites regarding their multivariate dispersion, pooling all periods together and within each period separately.**

|  | All periods | |  | End of wet season | |  | Early dry season | |  | Late dry season | |
| --- | --- | --- | --- | --- | --- | --- | --- | --- | --- | --- | --- |
|  | diff | p adj |  | diff | p adj |  | diff | p adj |  | diff | p adj |
| Anrumei - Abu | 0.001 | 1.000 |  | -0.044 | 0.799 |  | 0.034 | 0.446 |  | -0.024 | 0.920 |
| Anrumei - Bijante | -0.021 | 0.528 |  | -0.034 | 0.856 |  | 0.247 | 0.729 |  | -0.079 | 0.013 |
| Anrumei - Bruce | -0.034 | 0.084 |  | -0.090 | **0.037** |  | 0.009 | 0.995 |  | -0.039 | 0.575 |
| Anrumei - Escadinhas | -0.025 | 0.368 |  | 0.000 | 1.000 |  | 0.000 | 1.000 |  | -0.093 | **0.002** |
| Anrumei - Adonga | -0.098 | **<0.001** |  | -0.111 | **0.001** |  | -0.093 | **<0.001** |  | -0.125 | **<0.001** |
| Abu - Bijante | -0.022 | 0.491 |  | 0.009 | 1.000 |  | -0.009 | 0.996 |  | -0.055 | 0.191 |
| Abu - Bruce | -0.034 | 0.074 |  | -0.046 | 0.643 |  | -0.024 | 0.760 |  | -0.014 | 0.990 |
| Abu - Escadinhas | -0.025 | 0.336 |  | 0.044 | 0.681 |  | -0.033 | 0.423 |  | -0.069 | **0.045** |
| Abu - Adonga | -0.099 | **<0.001** |  | -0.067 | 0.129 |  | -0.126 | **<0.001** |  | -0.101 | **<0.001** |
| Bijante - Bruce | -0.012 | 0.907 |  | -0.055 | 0.222 |  | -0.015 | 0.951 |  | 0.040 | 0.444 |
| Bijante - Escadinhas | -0.003 | 1.000 |  | 0.035 | 0.712 |  | -0.025 | 0.711 |  | -0.014 | 0.989 |
| Bijante - Adonga | 0.079 | **<0.001** |  | -0.076 | **0.004** |  | -0.117 | **<0.001** |  | -0.046 | 0.288 |
| Bruce - Escadinhas | 0.009 | 0.973 |  | -0.090 | **0.005** |  | 0.009 | 0.995 |  | 0.054 | 0.160 |
| Bruce - Adonga | 0.065 | **<0.001** |  | -0.021 | 0.913 |  | -0.102 | **<0.001** |  | -0.087 | **0.002** |
| Escadinhas - Adonga | 0.074 | **<0.001** |  | -0.111 | **<0.001** |  | -0.093 | **<0.001** |  | -0.032 | 0.685 |
