# Supplementary material for: Spatial and seasonal variation in macrozoobenthic density, biomass and community composition in a major tropical intertidal area, the Bijagós Archipelago, West-Africa
Source: PLoS One. 2022 Nov 28;17(11):e0277861. doi: 10.1371/journal.pone.0277861 (PMC9704600; doi:10.1371/journal.pone.0277861)
Supplement: S6 Table — (DOCX) [file pone.0277861.s006.docx]

**Table S6: Post-hoc pairwise PERMANOVAs (with 100,000 permutations) comparing the macrozoobenthic community compositions between the sites within each period.**

|  | End of wet season | | |  | Early dry season | | |  | Late dry season | | |
| --- | --- | --- | --- | --- | --- | --- | --- | --- | --- | --- | --- |
|  | F.model | R2 | p adj |  | F.model | R2 | p adj |  | F.model | R2 | p adj |
| Anrumei - Abu | 1.140 | 0.049 | 1.000 |  | 3.395 | 0.047 | **0.008** |  | 1.940 | 0.040 | 0.663 |
| Anrumei - Bijante | 2.669 | 0.073 | 0.064 |  | 5.568 | 0.070 | **<0.001** |  | 6.537 | 0.116 | **<0.001** |
| Anrumei - Bruce | 6.229 | 0.159 | **<0.001** |  | 10.356 | 0.124 | **<0.001** |  | 8.964 | 0.152 | **<0.001** |
| Anrumei - Escadinhas | 2.855 | 0.077 | 0.054 |  | 6.454 | 0.080 | **<0.001** |  | 7.718 | 0.134 | **<0.001** |
| Anrumei - Adonga | 8.358 | 0.111 | **<0.001** |  | 7.701 | 0.125 | **<0.001** |  | 11.378 | 0.177 | **<0.001** |
| Abu - Bijante | 3.580 | 0.095 | **0.004** |  | 3.389 | 0.044 | **0.008** |  | 5.968 | 0.107 | **<0.001** |
| Abu - Bruce | 8.496 | 0.205 | **<0.001** |  | 5.678 | 0.073 | **<0.001** |  | 8.560 | 0.146 | **<0.001** |
| Abu - Escadinhas | 5.255 | 0.134 | **<0.001** |  | 3.691 | 0.048 | **0.003** |  | 8.964 | 0.152 | **<0.001** |
| Abu - Adonga | 10.170 | 0.132 | **<0.001** |  | 8.476 | 0.138 | **<0.001** |  | 16.863 | 0.241 | **<0.001** |
| Bijante - Bruce | 3.612 | 0.074 | **0.023** |  | 5.501 | 0.067 | **<0.001** |  | 7.180 | 0.117 | **<0.001** |
| Bijante - Escadinhas | 4.855 | 0.095 | **<0.001** |  | 4.111 | 0.050 | **<0.001** |  | 11.817 | 0.180 | **<0.001** |
| Bijante - Adonga | 19.435 | 0.197 | **<0.001** |  | 6.764 | 0.104 | **<0.001** |  | 20.659 | 0.266 | **<0.001** |
| Bruce - Escadinhas | 5.862 | 0.115 | **<0.001** |  | 5.934 | 0.072 | **<0.001** |  | 15.608 | 0.224 | **<0.001** |
| Bruce - Adonga | 34.066 | 0.304 | **<0.001** |  | 12.224 | 0.177 | **<0.001** |  | 23.423 | 0.291 | **<0.001** |
| Escadinhas - Adonga | 20.425 | 0.205 | **<0.001** |  | 10.786 | 0.157 | **<0.001** |  | 21.945 | 0.278 | **<0.001** |
