# Supplementary material for: Spatial and seasonal variation in macrozoobenthic density, biomass and community composition in a major tropical intertidal area, the Bijagós Archipelago, West-Africa
Source: PLoS One. 2022 Nov 28;17(11):e0277861. doi: 10.1371/journal.pone.0277861 (PMC9704600; doi:10.1371/journal.pone.0277861)
Supplement: S1 Fig — (DOCX) [file pone.0277861.s007.docx]

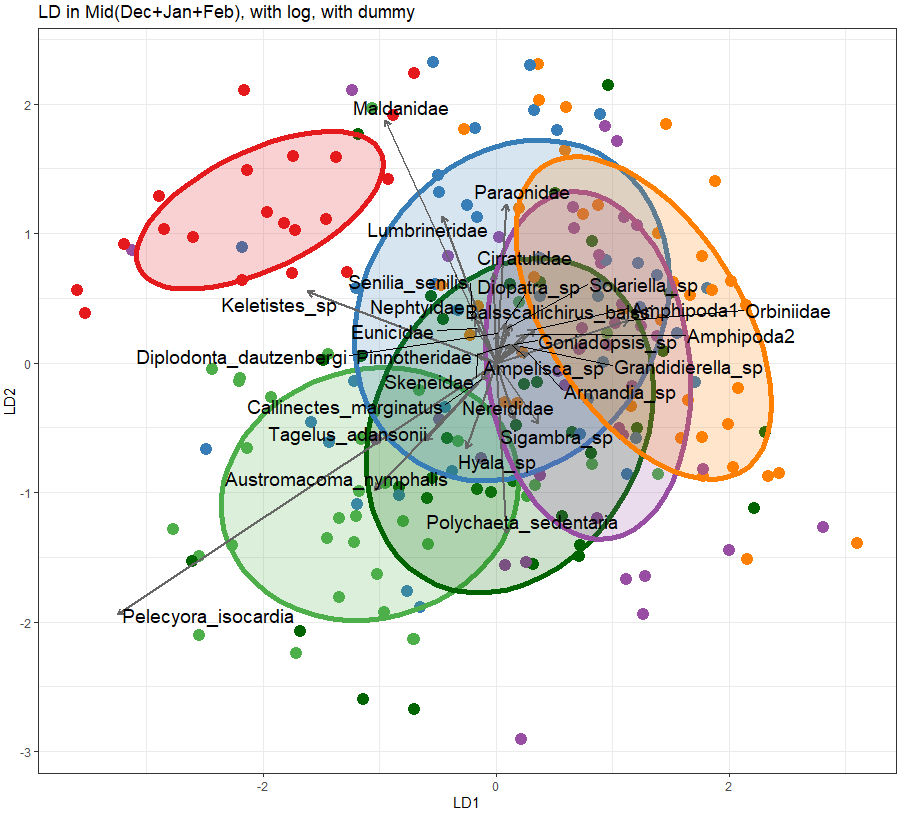

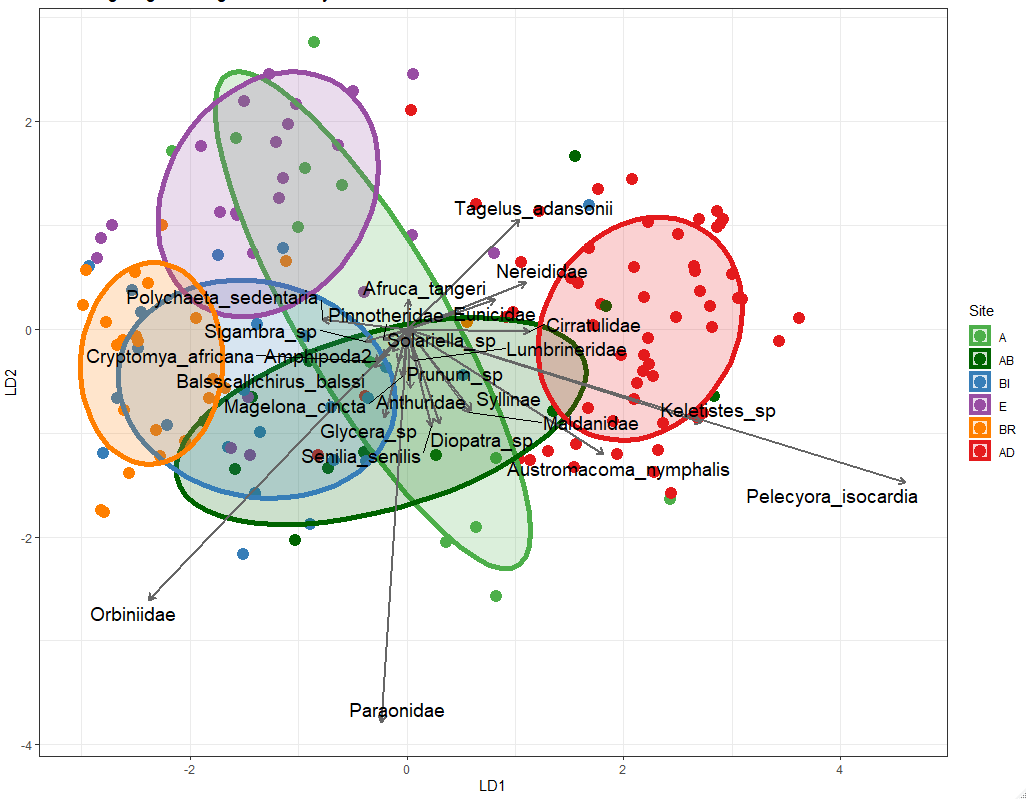

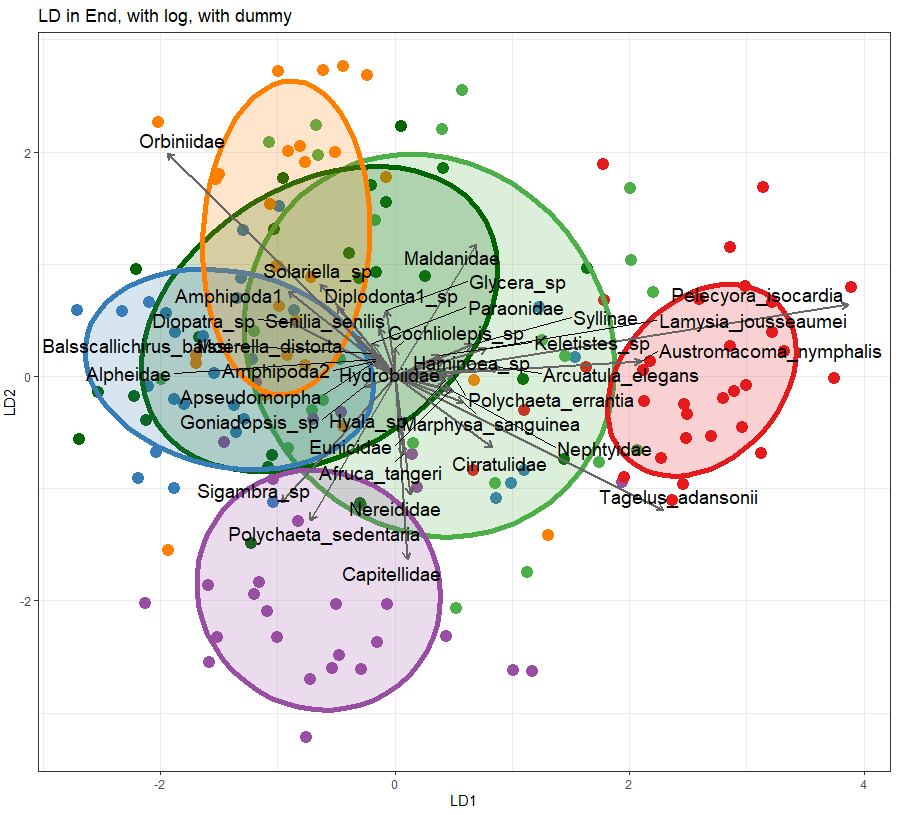

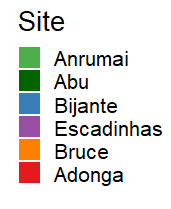

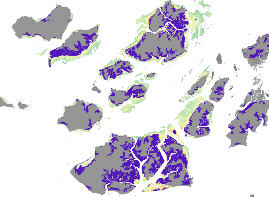


**Figure S1.** **CAP analysis based on discriminant analysis with all species displayed.** Canonical analysis of Principal Components based on discriminant analysis (made with 3 axis), grouping the data points (cores) per site, in the end of wet season (A: October – November); early dry season (B: December, January and February); and late dry season (C: March and April,) in a dual-axis linear discriminant space. Ellipses represent 60% of the data for each group. Arrows show species for which their abundances are significantly related to the axis of the ordination, and the size of the arrows is scaled according to the R^2^ of each regression (i.e., longer arrows represent more influent species).
