# Supplementary material for: Spatial and seasonal variation in macrozoobenthic density, biomass and community composition in a major tropical intertidal area, the Bijagós Archipelago, West-Africa
Source: PLoS One. 2022 Nov 28;17(11):e0277861. doi: 10.1371/journal.pone.0277861 (PMC9704600; doi:10.1371/journal.pone.0277861)
Supplement: S1 Appendix — (DOCX) [file pone.0277861.s008.docx]

**Appendix S1. Macrozoobenthos photographic reference collection**

https://zenodo.org/record/7255557
