# Supplementary material for: Spatial and seasonal variation in macrozoobenthic density, biomass and community composition in a major tropical intertidal area, the Bijagós Archipelago, West-Africa
Source: PLoS One. 2022 Nov 28;17(11):e0277861. doi: 10.1371/journal.pone.0277861 (PMC9704600; doi:10.1371/journal.pone.0277861)
Supplement: S2 Appendix — (DOCX) [file pone.0277861.s009.docx]

**Appendix S2. Spatial variation in species richness and diversity.**

Notes on methods

The spatial variation in species richness and Shannon-Wiener diversity index was explored by pooling sampling months, which vary to some extent with regards to sample size (Table S1 in Sup. Mat). As sites had different sample sizes (Table S1) and different densities of individuals, Shannon-Wiener and species richness were calculated by balancing the sampling effort both in relation to the number of cores and the number of individuals. The number of cores used for the calculations in each site was thus limited to 71 or less, based on the site with the lowest number of cores (Abu), and the total number of individuals in the selected cores for the analysis was limited to 800 in each site, to allow reaching the asymptote on the rarefication curves for every site (Figure 2 in Supp mat 2), while avoiding unbalanced number of individuals between sites. We calculated the Shannon-Wiener diversity index and species richness 1000 times by randomizing the cores used for the analysis, thus obtaining a mean and standard deviation from the bootstrapping.

**Table 1. Mean Shannon-Wiener, mean species richness and mean number of cores and of individuals used for the bootstrap calculations in each site, with standard deviations.**

| Site | Shannon-Wiener | | | Species richness | | | |
| --- | --- | --- | --- | --- | --- | --- | --- |
|  | **Mean ± SD** | **Mean**  **N. cores** | **Mean**  **N. ind.** | **Total** | **Mean ± SD** | **Mean**  **N. cores** | **Mean N. ind.** |
| Anrumai | 3.1 ± 0.1 | 60.5 ± 5.4 | 809.4 ± 10.6 | 51 | 44.6 ± 2.5 | 60.3 ± 5.3 | 810.2 ± 11.0 |
| Abu | 3.1 ± 0.1 | 53.1 ± 4.9 | 811.0 ± 8.4 | 51 | 44.7 ± 2.5 | 52.9 ± 4.7 | 811.4 ± 9.0 |
| Bijante | 3.2 ± 0.1 | 39.3 ± 3.6 | 814.3 ± 10.8 | 57 | 47.5 ± 2.1 | 39.2 ± 3.6 | 814.6 ± 10.6 |
| Escadinhas | 2.8 ± 0.1 | 69.6 ± 2.9 | 757.1 ± 52.1 | 44 | 37.4 ± 2.1 | 70.0 ± 2.7 | 758.0 ± 53.7 |
| Bruce | 3.0 ± 0.1 | 63.0 ± 5.0 | 806.9 ± 15.0 | 60 | 50.5 ± 2.8 | 63.0 ± 5.2 | 807.6 ± 12.9 |
| Adonga | 2.4 ± 0.1 | 28.9 ± 3.4 | 821.3 ± 18.8 | 52 | 35.4 ± 2.6 | 29.0 ± 3.5 | 820.8 ± 17.5 |

See methods for details. Total: maximum species richness in each site when considering all the cores sampled.

**B**

**A**


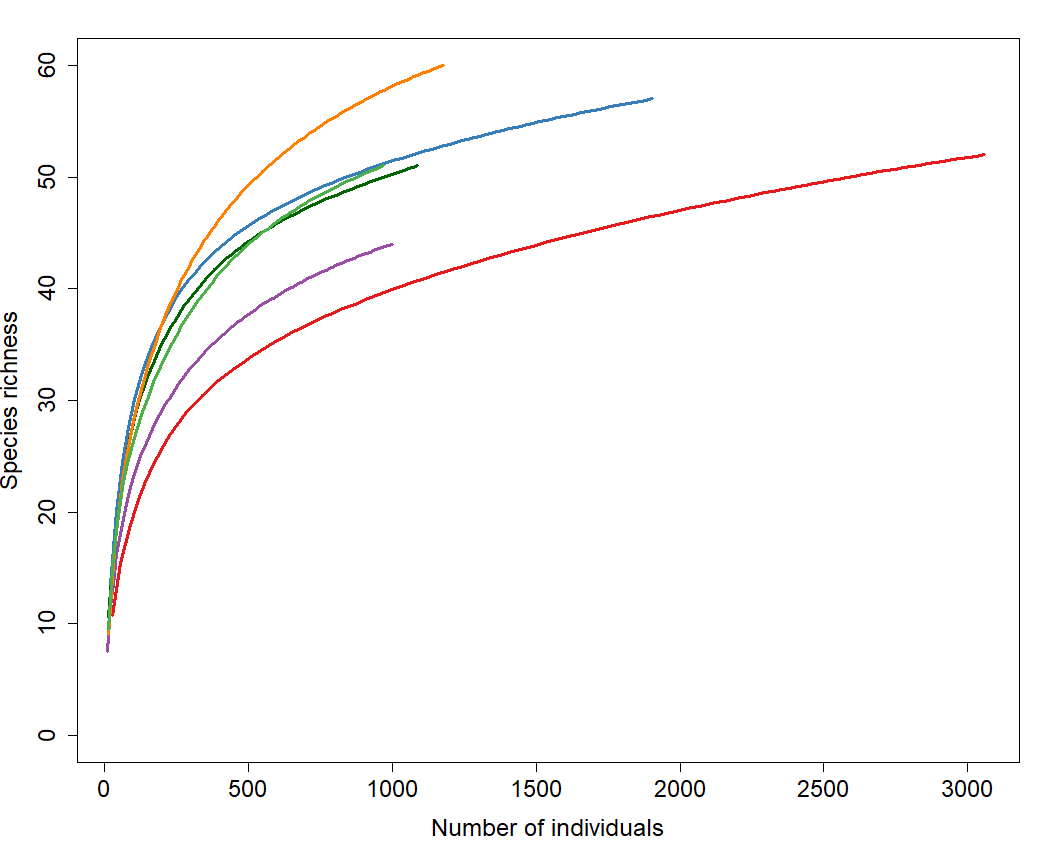

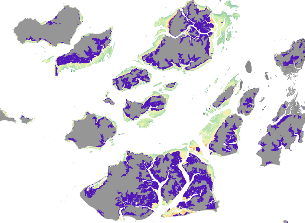

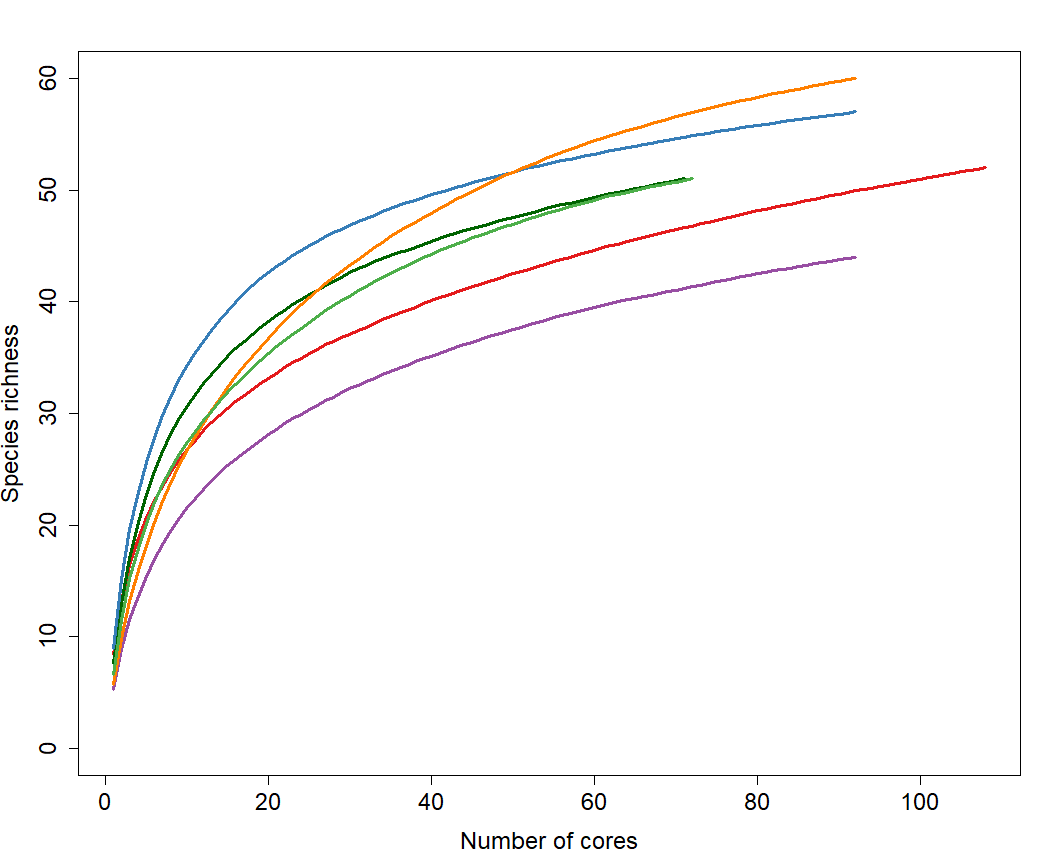

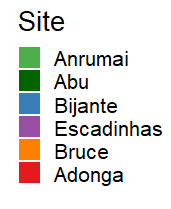


**Figure 1. Accumulation and rarefaction curves displaying the species richness with incremental sample sizes (A: cores, B: number of individuals) in each site.**
