# Supplementary material for: Spatial and seasonal variation in macrozoobenthic density, biomass and community composition in a major tropical intertidal area, the Bijagós Archipelago, West-Africa
Source: PLoS One. 2022 Nov 28;17(11):e0277861. doi: 10.1371/journal.pone.0277861 (PMC9704600; doi:10.1371/journal.pone.0277861)
Supplement: S1 Checklist — (DOCX) [file pone.0277861.s010.docx]

Inclusivity in global research

**Ethical considerations, permits and authorship**

*This section is applicable to all research types.*

Provide details as to who granted permissions and/or consent for the study to take place in the Methods section of your manuscript. This should include the names of **all** ethics boards, governmental organizations, community leaders or other bodies that provided approval for the study. If individuals provided approval refer to these people by their role or title but do not list their name(s).

The governmental organization responsible for biodiversity conservation in Guinea-Bissau (IBAP – Instituto da Biodiversidade e das Áreas Protegidas Dr. Alfredo Simão da Silva) and local organizations (NGOs), that have decades of experience working in the area with the local communities, were involved in the planning and realization of this study from the very onset. Permissions to sample on the study sites was formally granted by IBAP. In addition, and as written consent from the local community leaders is not possible in this area due to the very low education rates (writing is a rare skill), we attained verbal permission from local leaders to operate and sample on their lands on every study site.

If there were any deviations from the study protocol after approval was obtained please provide details of these changes in the Methods section of your manuscript.
Did this study involve local collaborators that are residents of the country where the research was conducted or members of the community studied? If you do not have any authors from said communities, please provide an explanation for this below.

Reported on page number: N/A

One of the first co-authors is a member of the local community where the study was conducted, and one of the authors of this study is an employee of the government institution for biodiversity and protected areas, Instituto da Biodiversidade e das Áreas Protegidas Dr. Alfredo Simão da Silva (IBAP). All the local collaborators that participated in field data collection and supported the logistics are acknowledged in the acknowledgments section.

Everyone listed as an author should meet PLOS’ criteria for authorship and all individuals who meet these criteria should be included in the author byline, rather than the acknowledgements. Authorship criteria is based on the International Committee of Medical Journal Editors (ICMJE) Uniform Requirements for Manuscripts Submitted to Biomedical Journals - for further information please see here: <https://journals.plos.org/plosone/s/authorship>.

**Human subjects research (e.g. health research, medical research, cross-cultural psychology)**

Did you obtain written informed consent from a representative of the local community or region before the research took place? How did you establish who speaks for the community? Details of written informed consent obtained from study participants should be reported separately in the Methods section of your manuscript.

N/A

How did members of the local community provide input on the aims of the research investigation, its methodology, and its anticipated outcome(s)?

Members of the local communities often accompanied the researchers in the field, joining them to collect sediment samples and sort the macrozoobenthos specimens together. Informal discussions between the authors and local people were held both during and after field data collection. Additionally, the local knowledge of the area shared by local community was key to define the sampling areas and ensure the best and most efficient coverage of the study area.

When engaging with the local community, how did you ensure that the informed consent documents and other materials could be understood by local stakeholders?

Authors of the paper were present in local meetings to explain the aims of the research to local leaders, while during field collection authors made sure to always explain the goals of the study in the local language.

Will the findings of the research be made available in an understandable format to stakeholders in the community where the study was conducted (e.g. via a presentation, summary report, copies of publications, etc.)? Please provide details of how this will be achieved.

This research was conducted within the framework of the project “Waders of the Bijagós - Securing

the ecological integrity of the Bijagos archipelago as a key site for waders along the East Atlantic Flyway”, that include partner institutions focused on communication and dissemination of the scientific findings of the project to local communities. Furthermore, authors have participated in several local events where they presented results of this study to community members.

**Non-human subjects research using specimens/ animals collected as part of the study, or those housed in archival collections. Examples include archaeology, paleontology, botany and zoology.**

Did the permission you obtained from a local authority to perform the study include an agreement on access to outputs and benefit sharing? This may include procedures to enable fair distribution of the benefits and resources arising from the research performed. Please include any details of Prior Informed Consent and Benefit Sharing Agreements obtained. These may be required by field-specific regulations, for example the Convention on Biological Diversity (CBD) and the associated Nagoya Protocol.

N/A

If the material used in your study was imported, please A) provide the year it was imported and B) indicate whether permits were obtained to import/export the materials used, C) provide details of any permits obtained. If this information is not available, please indicate this.

Permits to export macrozoobenthos samples from Guinea-Bissau to Portugal, where they were processed, were obtain from Instituto da Biodiversidade e das Áreas Protegidas Dr. Alfredo Simão da Silva, which was also a partner in this research project, for the years of 2018 to 2020.

If you used archival specimens, please state how the material used in your study was acquired by the institute it is held in and provide details of any permits obtained for the original excavations/ sample collection. If this information is not available, please indicate this.

N/A

How was the potential cultural significance of the materials collected in your study to local communities considered in your research design? Were Indigenous peoples and/or local researchers and institutions involved with archaeological excavations / collection of specimens? If so, please provide a description of their involvement.

One of the first co-authors of this study is a member of the local community and co-lead this research. The local institutions that collaborated with the authors of this study organized meetings to introduce the project to the local communities. Furthermore, macrozoobenthos samples were often collected together with local people that were harvesting shellfish, one of their main food sources, so a strong cultural interest in the study being developed was apparent. Authors always made sure to introduce themselves to the community leaders and openly discuss the goals and methodology of the study with them.

If your manuscript includes photographs of human remains please indicate whether authors obtained permission from descendants or affiliated cultural communities to do so.

N/A
